# Evolution of 3D-Printed Microneedles toward Closed-Loop Theranostic Platforms

**DOI:** 10.34133/research.1338

**Published:** 2026-07-21

**Authors:** Chuan Yang, Xinxin Yan, Yue Hou, Xiaolong Sun, Sahithi Lingala, Yang Yang, Ziyu Wang, Rui Xiong

**Affiliations:** ^1^ Key Laboratory of Artificial Micro- and Nano-structures of Ministry of Education, School of Physics and Technology, Wuhan University, Wuhan 430072, China.; ^2^ Department of Orthopedics, Renmin Hospital, Wuhan University, Wuhan 430072, China.; ^3^ School of Integrated Circuits, Wuhan University, Wuhan 430072, China.; ^4^ Department of Mechanical Engineering, San Diego State University, San Diego, CA 92182, USA.

## Abstract

Advancements in biomedical interfaces increasingly demand technologies that can seamlessly bridge the gap between biological tissues and therapeutic systems. Microneedle (MN) technology has emerged as a minimally invasive platform for transdermal drug delivery (TDD) and biosensing, offering tunable geometries, efficient skin penetration, and reduced patient discomfort. However, the inherent limitations of conventional microfabrication techniques in terms of structural complexity, multi-material compatibility, and functional modularization have markedly constrained the development of next-generation biomedical systems. In recent years, 3-dimensional (3D) printing has positioned itself as a highly promising additive manufacturing (AM) technology, offering exceptional design freedom and high-resolution fabrication capabilities for the development of MN platforms with embedded microchannels and integrated multifunctionality. Herein, a comprehensive analysis of recent progress in 3D-printed MNs is provided, with emphasis placed on advances in architectural innovations, intelligent system integration, and their expanding applications in personalized drug delivery and intelligent theranostic platforms. Furthermore, an in-depth examination of the core challenges hindering the clinical translation of 3D-printed MNs, particularly regarding manufacturing processes, material selection, and standardization requirements, is presented, offering a forward-looking perspective on the paradigm shift of MNs from passive delivery terminals to active, closed-loop health platforms.

## Introduction

Driven by the rapid advancement of biomedical technology [1], drug delivery systems (DDSs) are evolving toward enhanced efficiency, safety, and patient-centric care. While traditional oral and injectable routes remain standard, they are constrained by erratic bioavailability, lack of localized targeting, and the logistical burden of professional supervision in clinical settings, which often hinders patient compliance [2–4]. In recent years, microneedle (MN) technology has emerged as a transformative transdermal delivery and biosensing platform that bridges the gap between conventional injections and transdermal patches, demonstrating substantial potential for clinical and diagnostic applications [5–7]. Structurally, MNs consist of micrometer-scale projections capable of breaching the stratum corneum, which serves as the skin’s primary barrier. This mechanism circumvents the low permeability of the stratum corneum that limits the transport of macromolecules while creating temporary, minimally invasive conduits that typically avoid major nerves and blood vessels in the dermis. This unique approach not only facilitates painless, highly efficient substance delivery but also has the potential to enable patients with the capacity for at-home self-administration [8–10]. By eliminating the need for clinical infrastructure and reducing infection risks, MNs represent a critical shift toward decentralized, personalized healthcare, offering a superior balance between therapeutic efficacy and user-friendliness [11–13].

**Fig. 1.**
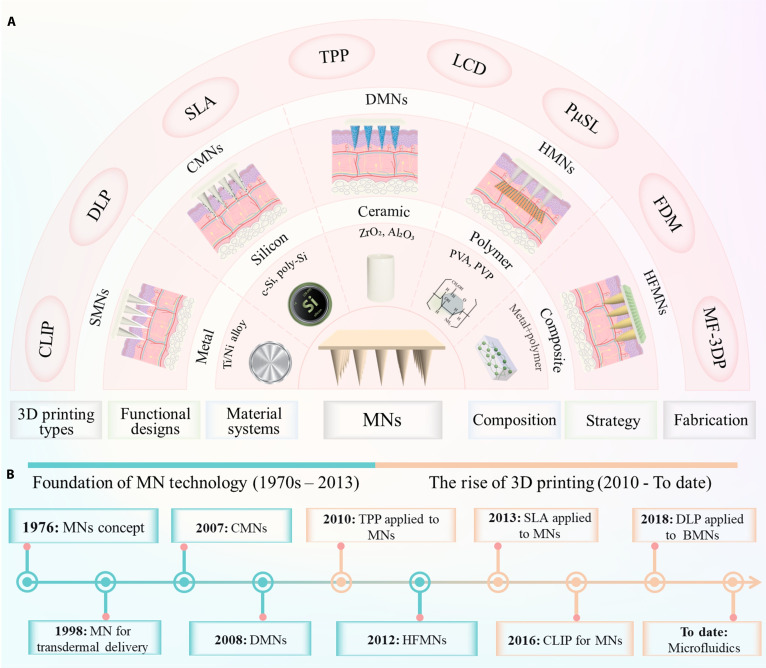
The roadmap of microneedle (MN) technology. (A) Overarching framework of MN technology. (B) Developmental trajectory of MN systems from conceptual inception to intelligent integration. CMNs, coated microneedles; DMNs, dissolving microneedles; HFMNs, hydrogel-forming microneedles; BMNs, bioinspired microneedles; SLA, stereolithography; CLIP, continuous liquid interface production; DLP, digital light processing; TPP, two-photon polymerization; LCD, liquid crystal display; PμSL, projection micro-stereolithography; FDM, fused deposition modeling; MF-3DP, magnetic field-assisted 3D printing; PVA, polyvinyl alcohol; PVP, polyvinylpyrrolidone; c-Si, crystalline silicon; poly-Si, polycrystalline silicon.

The evolution of MN technology has undergone a multi-stage development process, ranging from material exploration and structural iteration to intelligent functional integration systems (Fig. 1). Early MNs were primarily based on inorganic materials, such as silicon and metals, fabricated using micro-electromechanical system (MEMS) technology [14,15]. Despite their excellent mechanical properties, widespread application was impeded by high manufacturing costs, complex fabrication processes, and potential biocompatibility issues. With continuous progress in polymer science, polymeric MNs, particularly dissolvable and hydrogel types, have accelerated clinical translation due to their biodegradability, controllable release capabilities, and elimination of sharps waste [16–18]. More recently, MN technology has undergone a paradigm shift from passive delivery vehicles to intelligent theranostic platforms. By synergizing advanced functional materials with flexible bioelectronics, emerging MN systems, ranging from stimuli-responsive arrays to wearable patches, have achieved the seamless integration of drug delivery, biofluid sampling, and multimodal signal monitoring [19–22]. Figure 1B highlights a parallel shift in fabrication paradigms—from the geometric constraints of conventional MEMS and molding workflows to digital additive manufacturing (AM), exemplified by 3-dimensional (3D) printing. By expanding design freedom (such as bioinspired barbs and embedded microfluidic channels), AM accelerates the iteration of MN architectures and supports personalized, multifunctional biomedical systems.

**Fig. 2.**
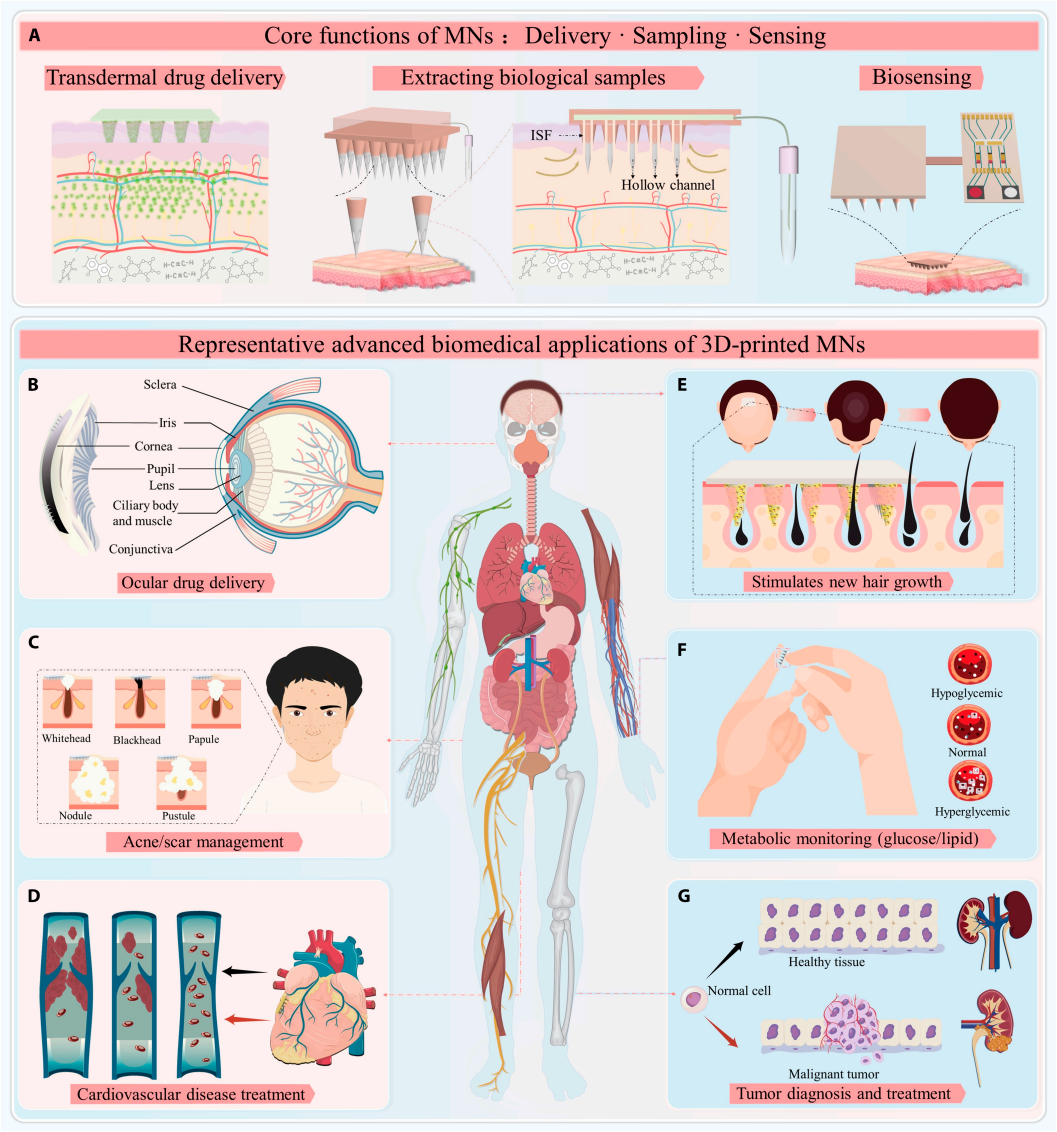
Core functions and advanced biomedical applications of 3D-printed MNs. (A) Three principal functions: extracting biological samples, TDD, and biosensing. (B to G) Representative applications of 3D-printed MNs in ocular drug delivery, acne/scar management, hair regeneration, metabolite monitoring, cardiovascular disease therapy, and tumor diagnosis and treatment.

Despite thesubstantial progress of MN technology, conventional fabrication methods such as micro-injection molding and photolithography still face critical challenges, including complex procedures, high production costs, and limited design flexibility [23,24]. These constraints hinder the ability to meet the growing demand for personalized medicine and complex functional integration. Against this backdrop, the emergence of 3D printing technology has introduced a transformative paradigm shift in the MN field. By leveraging its high degree of structural design freedom, broad material compatibility, and precise micro-scale fabrication capabilities, 3D printing has not only addressed many of the limitations associated with traditional manufacturing but also greatly expanded the potential for structural innovation, functional integration, and personalized customization [25–28]. As illustrated in Fig. 2, 3D-printed MNs are capable of performing fundamental functions such as TDD, biofluid extraction, and biosensing (Fig. 2A) while also being applied across a wide array of cutting-edge biomedical fields. These applications span a spectrum from superficial interventions (such as acne and scar treatment and hair regeneration) to deep-tissue regulation (such as ocular drug delivery and cardiovascular therapy) and systemic disease management (such as metabolite monitoring, tumor diagnosis, and treatment) (Fig. 2B to G) [28–30]. Such versatility fully demonstrates the immense value of 3D-printed MNs as an advanced platform technology for developing integrated systems for diagnosis and therapy.

While substantial progress has been made in general MN fabrication and material selection, a critical gap remains in systematically connecting high-fidelity 3D-printed structural topologies with active, bioelectronic, and microfluidic closed-loop operations. Addressing this gap is essential to overcome the long-standing limitations of conventional MNs, particularly regarding structural monotony, functional fragmentation, and a lack of personalization. Capitalizing on 3D printing-enabled geometric freedom facilitates a profound paradigm shift from traditional passive delivery terminals toward active, mechanism-driven intelligent health platforms. Consequently, this review charts the technological evolution of 3D-printed MNs toward integrated closed-loop theranostic platforms. It covers manufacturing techniques, material selection, bioinspired structural optimization strategies, and the leveraging of advanced 3D printing modalities. Subsequently, frontier biomedical applications are systematically analyzed, categorized into 2 distinct domains: standalone functional patches for multi-dimensional therapeutics, ranging from skin barrier remodeling to systemic regulation and tumor immunotherapy, and integrated intelligent systems for bioelectronic diagnostics and active feedback interventions. Finally, the challenges impeding clinical translation are critically examined, and the future trajectory toward closed-loop theranostic platforms is outlined.

## Fundamentals of MN Technology

### Classification and therapeutic mechanisms

**Fig. 3.**
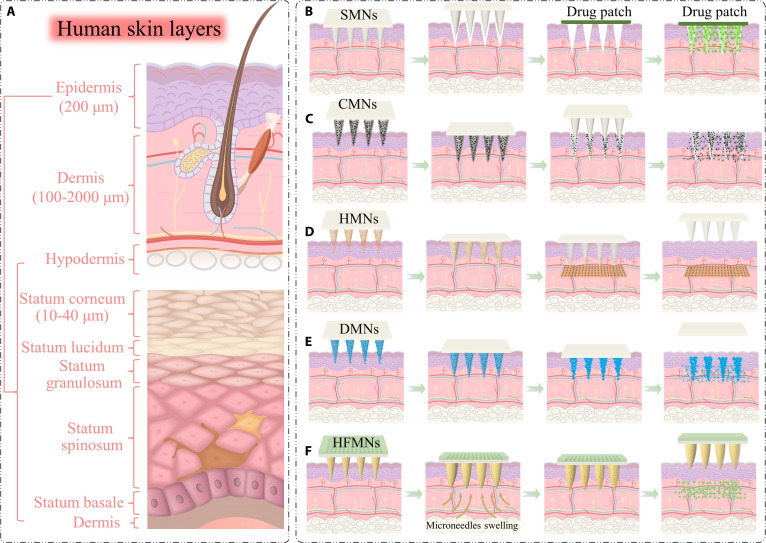
Schematic illustration of MN structural types and their mechanisms of action. (A) Anatomical layers of the skin penetrated by MNs, including the epidermis, dermis, and subcutaneous tissue. (B) SMNs [40–42]. (C) CMNs [43–45]. (D) HMNs [46–48]. (E) DMNs [49–51]. (F) HFMNs [52–54].

As illustrated in Fig. 3A, human skin primarily consists of 3 layers: the epidermis, dermis, and hypodermis. The stratum corneum, which constitutes the outermost layer of the epidermis, serves as the primary physical barrier to transdermal drug delivery (TDD) and comprises 10 to 20 layers of nonviable corneocytes, forming a layer approximately 10 to 40 μm thick [31–34]. Beneath this barrier, the viable epidermis and dermis are richly vascularized, rendering them ideal target sites for drug absorption. MN technology creates micrometer-scale channels in the skin through a physical mechanism, bypassing the stratum corneum and enabling the direct delivery of therapeutic agents into the deeper epidermis or superficial dermis. This markedly enhances drug permeation efficiency while offering a painless or minimally painful administration strategy [35,36].

Based on their structural characteristics, material properties, and delivery mechanisms, current MN systems are generally categorized into solid MNs (SMNs), coated MNs (CMNs), hollow MNs (HMNs), dissolving MNs (DMNs), and hydrogel-forming MNs (HFMNs) (Fig. 3B to F) [5,24,37–39]. Each type exhibits distinct functional features and offers unique advantages, making them particularly suitable for specific clinical and research applications. SMNs represent the most fundamental and extensively studied category, typically fabricated from materials like metals and silicon with superior mechanical strength to perform a “poke and patch” approach [40–42]; however, this 2-step procedure can be clinically inconvenient and leads to pharmacokinetic variability. CMNs follow a “coat and poke” model, where a water-soluble formulation is coated onto the needles and rapidly dissolves upon contact with interstitial fluid (ISF) [43–45], although drug loading is limited by the MN surface area. HMNs feature internal hollow channels for a “poke and flow” mechanism, enabling precise control over larger drug volumes and administration rates, or even ISF collection [46–48]. To eliminate sharp waste and improve safety, DMNs are composed of biodegradable polymers [such as hyaluronic acid (HA) and polyvinyl alcohol (PVA)] that serve as both the carrier and the needle body, releasing drugs via a “poke and dissolve” model [49–51]. HFMNs represent an emerging class that swells upon ISF absorption to form a continuous hydrogel channel, providing near-zero-order release kinetics for prolonged delivery [52–54]. A comparative summary of these systems is provided in Table 1.

**Table 1.** Classification and characteristics of predominant MN systems

MN type	Delivery mechanism	Key advantages	Main disadvantages	Application scenarios	Ref.
SMNs	Poke and patch	Simple structure, mature fabrication technology, superior mechanical properties	Two-step operation, drug delivery efficiency depends on the subsequent patch	Skin pretreatment, enhancing transdermal absorption	[40–42]
CMNs	Coat and poke	Simple operation, rapid drug delivery	Low drug loading, complex coating process	Vaccine delivery, scenarios requiring rapid onset of action (low-dose, high-potency formulations)	[43–45]
HMNs	Poke and flow	High drug loading capacity, precise control over dose and flow rate	Reliance on external pumps, needle clogging, complex manufacturing	High-volume infusion, pulsatile drug delivery, interstitial fluid (ISF) extraction	[46–48]
DMNs	Poke and dissolve	Biodegradable, enhanced safety, relatively high drug loading	Limited mechanical strength, drug stability sensitive to temperature/humidity	Vaccination, chronic disease management	[49–51]
HFMNs	Poke and release	Excellent biocompatibility, capable of sustained release	Slow drug release rate, structural stability may deteriorate after swelling	Long-term drug delivery, biosensing/monitoring	[52–54]
PMNs	Capillary action/Self-driven	Enables efficient, self-driven drug loading and sampling	Complex fabrication, potential for pore blockage	Sustained drug delivery, biosensing	[55,56]
CSMNs	Layered dissolution/Programmed release	Enables multi-drug and programmed release	Complex fabrication process	Multi-stage or targeted drug release	[57,58]
CryoMNs	Melt and release	Suitable for thermosensitive drugs, leaves no sharp residue	Requires robust cold-chain storage and transportation	Delivery of proteins, peptides, and other biologics	[59,60]

In recent years, MN technology has extended beyond traditional classifications to meet diverse clinical needs. For example, porous MNs (PMNs) feature interconnected porous networks for spontaneous drug loading and efficient ISF extraction [55,56]. Core–shell MNs (CSMNs) encapsulate different functional materials within distinct layers to allow for multi-stage or targeted release [57,58]. Another emerging system is cryomicroneedles (CryoMNs), which remain solid at low temperatures and rapidly melt upon insertion to deliver thermosensitive biologics without residue [59,60]. Finally, bioinspired MNs (BMNs), mimicking biological structures like animal stingers or insect mouthparts, show immense potential in improving operational convenience and penetration efficiency. Distinct from the functional classifications above, BMNs represent a morphology-driven innovation that has been greatly empowered by advanced manufacturing technologies, particularly 3D printing. A detailed discussion of 3D-printed BMNs is provided in the “Structural realization of complex 3D-printed BMNs”.

### Material selection

As a minimally invasive biomedical tool, the performance and safety of MNs are critically dependent on the physicochemical properties of the materials used in their fabrication. An ideal MN material should possess adequate mechanical strength to ensure effective penetration of the stratum corneum without fracturing or deforming while also exhibiting excellent biocompatibility to minimize adverse local tissue responses. Furthermore, material characteristics such as biodegradability, chemical stability, and processability are also critical considerations, depending on specific application requirements. Currently, MN materials are primarily categorized into 2 main classes: inorganic materials and polymeric substances, each exhibiting distinct advantages and limitations in practical applications [41,61–74]. Inorganic materials, encompassing silicon, glass, metals, and ceramics, were among the earliest employed due to mature micro-etching techniques and superior mechanical robustness [41,61–68]. However, the intrinsic brittleness of silicon and glass considerably increases the risk of fracture [41,61,62], while the potential release of metal ions from alloys [63–65] and the complex processing requirements of ceramics [66–68] limit their widespread adoption. Consequently, polymeric materials have emerged as the dominant choice due to their excellent design versatility and controllable degradation [69–74], ranging from nondegradable polymers [such as polycarbonate (PC) and polymethyl methacrylate (PMMA)] to biodegradable substances [such as polylactic acid (PLA), PVA, and HA]. Recently, composite materials integrating functional components like carbon nanotubes, graphene, or silk fibroin (SF) have enabled synergistic optimization of mechanical performance and the realization of advanced functionalities such as biosensing and smart drug delivery [75–80]. In summary, the evolution from early inorganic materials to modern multifunctional polymers and composites reflects a progressive shift toward safer, more efficient, and intelligent MN systems. In practical development, it is essential to balance mechanical properties, biocompatibility, degradation behavior, and manufacturing costs to achieve optimal design and facilitate successful industrialization of MN products. Crucially, achieving this intricate balance between material behavior and biological compatibility remains fundamentally bound to the geometric precision of the final device. Traditional casting or molding workflows inherently falter at preserving the structural fidelity of these advanced architectures, underscoring the absolute necessity of high-resolution AM for true functional realization.

## 3D Printing Techniques for MN Fabrication

### From traditional fabrication to 3D printing

With the continuous expansion of MN material systems, the ability to efficiently, precisely, and controllably fabricate these materials into desired structures has become a critical bottleneck. Conventional MN fabrication processes, largely derived from MEMS platforms, utilize techniques such as photolithography, micro-milling [81], micro-molding [82,83], laser beam machining (LBM) [84,85], and injection molding [24,86] to accommodate different material configurations. While these methods have advanced the field, they inherently suffer from limitations such as complex, multi-step workflows, high mold dependency, and inadequate batch-to-batch consistency. For instance, micro-milling is hindered by tool wear, while micro-molding and injection molding face challenges in high-fidelity replication of complex microstructures and limited mold lifespan. These constraints create a pressing need for next-generation fabrication technologies that offer enhanced design flexibility and rapid prototyping capabilities. In this context, 3D printing, leveraging the principles of AM, has emerged as a transformative direction for customized, high-throughput, and multifunctional MN production. A comprehensive comparison of the key characteristics between these conventional micromanufacturing techniques and 3D printing methods is summarized in Table 2.

**Table 2.** Comparative analysis of MN manufacturing technologies

Manufacturing method	Principle	Structural design freedom	Material suitability	Cost	Process complexity	Customization capability	Ref.
Micro-milling	Subtractive manufacturing	Low	Narrow	High	High	Low	[81]
Micro-molding	Replicative molding	Medium	Narrow	High	High	Low	[82,83]
LBM	Subtractive manufacturing	Medium	Narrow	High	Medium	Medium	[84,85]
Injection molding	Replicative molding	Low	Narrow	High	High	Low	[24,86]
3D printing	Additive manufacturing	Highest	Wide	Low	Low	High	[28–30]

3D printing, also known as AM, is an advanced fabrication technology that constructs physical structures by sequentially depositing materials layer by layer based on digital models [87–89]. Compared with conventional subtractive or formative manufacturing methods, 3D printing adopts a bottom-up fabrication approach, eliminating the need for molds and predefined toolpaths. This shift from “manufacturing-limited design” to design-driven manufacturing fundamentally reshapes production concepts, enabling the high-fidelity transformation of virtual designs into functional structures while expanding application boundaries across diverse sectors [89–92]. Its core strength lies in enabling mold-free fabrication, greatly reducing the development cost and cycle time for products with complex structures, making it particularly suitable for small-batch, customized rapid prototyping and iterative design with trial-and-error [93–95]. More importantly, this topological freedom facilitates seamless structure–function integration, enabling the efficient fabrication of intricate internal architectures, freeform surfaces, and multi-scale porous structures that were previously impossible to produce monolithically [94–97].

With respect to material versatility, modern 3D printing technologies have surpassed their initial limitations to basic photosensitive resins or thermoplastics, now encompassing a broad range of biodegradable polymers, hydrogels, metal alloys, ceramics, and even biofunctional materials capable of cell encapsulation or drug loading. Furthermore, multi-material cofabrication within a single printing process has become increasingly feasible, paving the way for the monolithic construction of heterogeneous architectures and enabling the creation of smart devices with compositional gradients and functional heterogeneity [98–101]. Additionally, by depositing material only where it is necessary, 3D printing eliminates several traditional manufacturing steps, such as mold fabrication, complex machining, and assembly, thereby substantially shortening the design-to-production timeline. Currently, 3D printing is widely applied across diverse sectors, including aerospace, automotive engineering, biomedical device manufacturing, architecture, education, and creative industries, demonstrating strong cross-sector adaptability and innovation potential [102–105]. In conclusion, 3D printing has emerged as a transformative force in advanced manufacturing, offering unique advantages in design flexibility, material integration, fabrication efficiency, and digital-driven production. Its ongoing advancements in printing resolution, build speed, and material diversity provide robust and scalable solutions to longstanding challenges in micro-scale device manufacturing. These characteristics make 3D printing particularly well-suited for the fabrication of MN systems, which demand high precision, structural complexity, and specific biological functionalities.

### Classification of 3D printing modalities

Compared to traditional methods such as micro-injection molding and etching, which are often constrained by resolution, material adaptability, process complexity, design flexibility, and cost control [24,82,85,86], 3D printing offers a versatile platform for the high-precision fabrication of structurally complex and personalized MN arrays [25,26,94]. Currently, 3D printing technologies applied to MN manufacturing are primarily categorized into vat photopolymerization (VPP)-based technologies and extrusion-based and functionalized 3D printing [106–121]. The strategic selection among these techniques is dictated by a multi-dimensional trade-off between structural resolution, manufacturing throughput, and material functionalization.

#### VPP-based technologies

VPP-based technologies represent the most prevalent high-precision manufacturing strategies for MNs. This category includes diverse processes such as stereolithography (SLA), continuous liquid interface production (CLIP/iCLIP), digital light processing (DLP), projection micro-stereolithography (PμSL), two-photon polymerization (TPP), and liquid crystal display (LCD) printing.

##### SLA

**Fig. 4.**
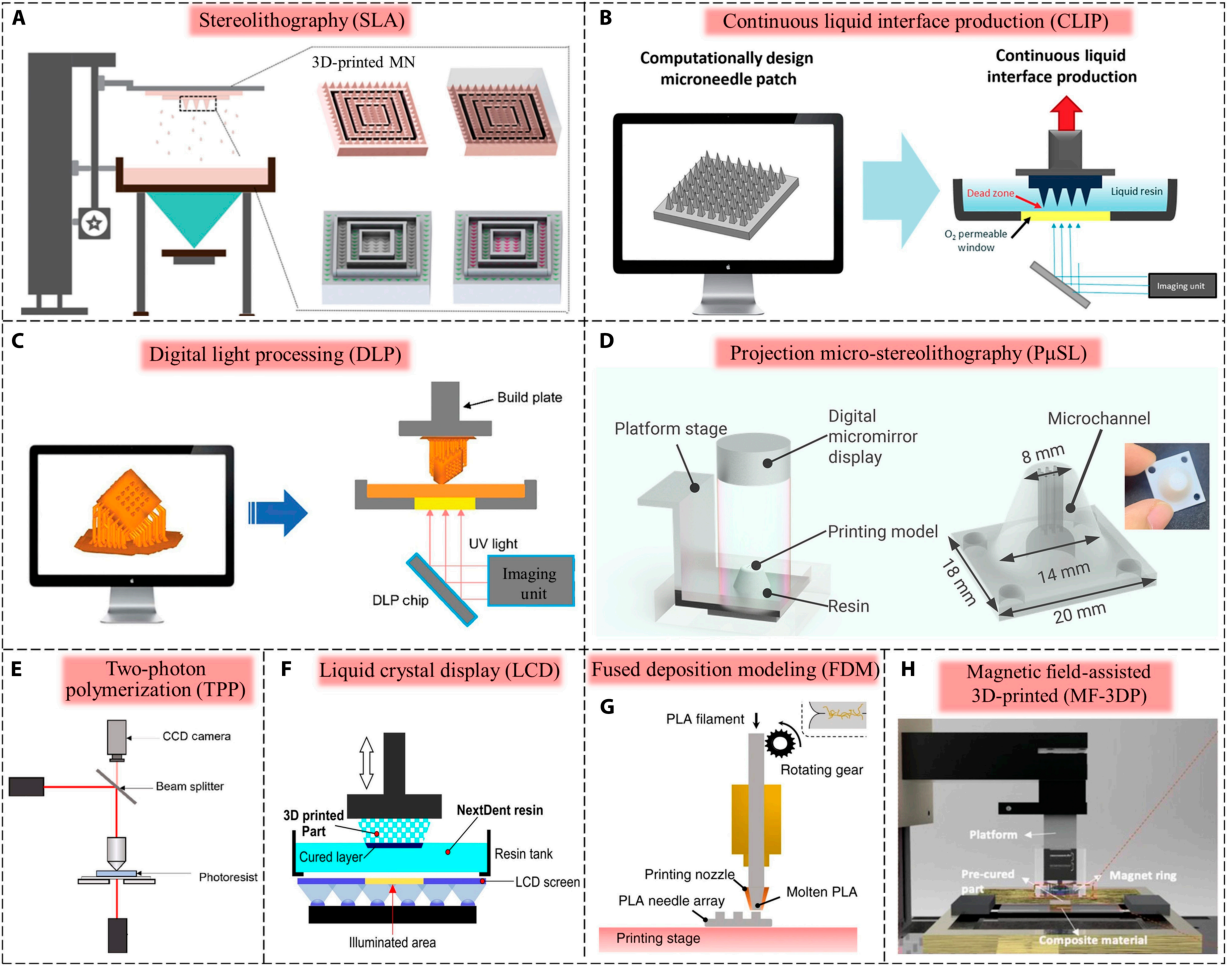
Principal manufacturing techniques for 3D-printed MNs. (A) SLA. Reproduced with permission from [106]. Copyright 2020, Wiley-VCH. (B) CLIP. Reprinted from [108] under a CC BY 4.0 license. (C) DLP. Reproduced with permission from [111]. Copyright 2024, Wiley-VCH. (D) PμSL. Reproduced with permission from [113]. Copyright 2024, Cell Press. (E) TPP. Reprinted from [114] under a CC BY 4.0 license. (F) LCD. Reproduced with permission from [116]. Copyright 2021, Elsevier. (G) FDM. Reprinted from [118] under a CC BY 4.0 license. (H) MF-3DP. Reproduced with permission from [120]. Copyright 2021, Wiley-VCH.

As a pioneering VPP technique, SLA achieves micrometer-level resolution by utilizing an ultraviolet (UV) laser beam to precisely scan and cure liquid photopolymer resin layer-by-layer (Fig. 4A) [106]. MN arrays fabricated using SLA exhibit smooth surfaces and high structural precision, making it particularly suitable for prototyping and validation stages. However, due to its vector-scanning mode, SLA exhibits relatively slow printing speeds and limited material options, being restricted to specific photopolymer resins.

##### CLIP

Based on the principles of SLA, CLIP introduces an oxygen-permeable window at the bottom of the resin vat to create a “dead zone”, which inhibits photopolymerization and enables a continuous, rapid printing process (Fig. 4B) [108]. It offers high resolution, mold-free forming, and the capacity to manufacture complex structures, notably enhancing manufacturing efficiency. Its advanced variant, injection continuous liquid interface production (iCLIP), further solves the problem of over-curing caused by residual resin in internal cavities. By actively injecting resin, it enables the integrated formation of MNs and microchannel structures, thereby offering a novel solution for multifunctional drug carriers [109].

##### DLP

Unlike SLA, DLP employs a mask-projection method to cure entire layers simultaneously using a UV light pattern projected by a digital micromirror device (DMD) (Fig. 4C) [111]. This technique substantially increases the printing speed, making it suitable for the batch production of complex structures like HMNs. DLP maintains high resolution while offering enhanced material compatibility, expanding the possibilities for functionalized MN applications. The further developed PμSL integrates a high-resolution projection system with a precision motion platform, achieving submicrometer printing accuracy and making it an ideal technique for fabricating high-resolution medical MNs and microfluidic chips (Fig. 4D) [113]. PμSL achieves an excellent balance between precision and speed, making it a preferred choice for manufacturing high-precision medical MNs and microfluidic chips.

##### TPP

In the pursuit of ultimate precision, TPP represents the forefront of micro-nano fabrication. TPP utilizes an ultrafast laser to induce 2-photon absorption, enabling resin curing exclusively at the focal point, which allows for the construction of nanoscale precision and complex internal structures (such as hollow channels and microfluidic pathways) within MNs (Fig. 4E) [114]. The resulting products possess excellent mechanical strength and tip sharpness, making them suitable for ultra-precision drug delivery applications, including ocular and inner-ear treatments. However, TPP is hampered by low printing efficiency and high equipment costs, which currently restrict its application primarily to high-end scientific research and the fabrication of high-value-added products.

##### LCD

Compared to the aforementioned technologies, this approach utilizes an LCD as a UV mask, achieving a favorable balance between cost, resolution, and speed (Fig. 4F) [116]. Studies have demonstrated that HMNs fabricated via LCD printing can be surface-modified for the transdermal delivery of peptide drugs, underscoring its potential in personalized medicine. However, it should be noted that the LCD panel is a consumable component with a finite lifespan, and its light intensity uniformity and printing accuracy are somewhat inferior to those of DLP.

#### Extrusion-based and functionalized 3D printing

Beyond VPP, other modalities provide unique advantages for specific material and functional requirements, primarily featuring fused deposition modeling (FDM) and magnetic field-assisted 3D printing (MF-3DP).

FDM: FDM operates by melting and sequentially depositing thermoplastic materials through a heated nozzle (Fig. 4G) [118]. Its advantages include the use of biocompatible materials, low equipment costs, and widespread accessibility, making it a cost-effective option for the industrial-scale production of MNs. However, FDM suffers from relatively low resolution, making it difficult to directly fabricate sharp tips. As a result, FDM-printed MNs often require post-processing, such as chemical etching, to achieve desirable tip geometries. Moreover, interlayer adhesion may be weak, posing risks of delamination during post-processing, thus necessitating optimization of printing parameters.

MF-3DP: This innovative approach applies an oriented magnetic field during photopolymerization to guide the alignment of magnetic nanoparticles within the polymer matrix (Fig. 4H) [120]. This technique markedly enhances the mechanical strength and penetration capability of MNs by constructing biomimetic reinforcement structures. However, it imposes higher material requirements, necessitating the incorporation of magnetic particles, and requires precise coordination between the magnetic field and the photopolymerization process, which increases equipment complexity.

In conclusion, these advanced 3D printing technologies have greatly propelled the field of MN manufacturing forward. They enable the high-precision fabrication of structurally complex MNs and provide a solid technical foundation for their functionalization and personalized design. Depending on specific demands for resolution, speed, biocompatibility, and cost in practical applications, researchers can flexibly select the most suitable technique to overcome the limitations of traditional manufacturing methods. With ongoing advancements in materials and printing technologies, 3D printing is poised to play an increasingly pivotal role in the MN field, propelling TDD technology to new frontiers.

### Structural realization of complex 3D-printed BMNs

Bioinspired design strategies, drawing inspiration from diverse biological systems, have emerged as a novel pathway for comprehensively enhancing the integrated performance of MNs. The core of bioinspired design lies in the systematic analysis of intrinsic mechanisms linking structure, function, and behavior in living organisms, extracting evolutionary “design blueprints”, and translating them into innovative engineering solutions [122–124]. Within the field of MNs, bioinspired strategies have evolved beyond superficial mimicry of biological morphology, progressing toward an in-depth exploration of synergistic structure–function relationships. This approach has substantially augmented the overall performance of MN systems in areas such as efficient skin penetration, robust adhesion, low-trauma insertion, and smart responsive transport [125–127]. Microscopic structures found in nature, such as insect mouthparts, animal spines, and plant trichomes, exhibit highly optimized configurations and mechanical properties, granting them superior capabilities for penetration, adhesion, fluidic transport, and environmental sensing [128–133]. Inspired by these biological principles, BMNs demonstrate notable advantages in penetration efficiency, mechanical stability, tissue conformability, pain minimization, and the integration of diagnostic and therapeutic functionalities. These attributes establish a robust technological foundation for their application in fields such as personalized medicine, intelligent drug delivery, and continuous biosignal monitoring.

While bioinspired strategies offer evolutionary “design blueprints” for enhancing MN performance, their clinical translation has long been hindered by the limitations of traditional manufacturing. Traditional manufacturing techniques, such as micro-milling, micro-injection molding, and mold forming, often face limitations, including challenges in mold release, insufficient resolution, complex fabrication processes, and high costs, especially when applied to designs with complex curvatures, internal cavities, multi-scale integrations, and nonuniform arrays. Moreover, these methods often struggle to realize highly intricate biomimetic topologies [24,82,85,86]. In contrast, as a bottom-up, layer-by-layer AM technology, 3D printing has successfully broken through the inherent geometric constraints of traditional subtractive manufacturing processes, providing strong support for the rapid prototyping and precision fabrication of BMNs. Its digital fabrication paradigm allows for the high-fidelity reconstruction of complex configurations directly from computer-aided design models, making it particularly suitable for replicating intricate features such as barbed insect mouthparts and gradient-stiffness plant spines. Furthermore, 3D printing offers unique advantages in the construction of complex curved surfaces, internal channels, and the integration of functional materials, thereby considerably enhancing MNs’ tissue adaptability, mechanical performance, and smart drug delivery capabilities [134–145]. A comparison of the fabrication strategies and functions of these 3D-printed BMNs is presented in Table 3.

**Table 3.** A comparison of fabrication strategies and functions of 3D-printed BMNs

Inspiration	Mechanism	MN type	Structural features	3D printing method	Application	Ref.
Blue-ringed octopus	Thermosensitive injection, wet adhesion	Degradable thermosensitive HFMNs	Conical array	PμSL	Oral ulcers, early-stage melanoma	[138]
Crab claw	Enhanced grip, reduced insertion force	Deformable HFMNs	Inclined needle body, surface microgrooves, kirigami structure	3D-printed mold	Tissue regeneration in diabetic ulcers	[136]
Mosquito’s labrum	Reduced insertion force, efficient penetration	HMNs	Labrum-like tip, 50-μm radius of curvature	SLA	Broad-spectrum antibiotic transdermal delivery	[140]
Sea cucumber	Hydrophilic/conductive/piezoelectric/anchoring	SMNs	Outer MN layer, inner parallel microchannels	PμSL	Peripheral nerve repair, inhibition of muscle atrophy	[142]
Coral	Microporous siphon, mechanical reinforcement	PMNs	Surface micropores and diversion grooves,	DLP	Chronically infected wounds	[143]
Succulent	Humidity-responsive shape morphing and adhesion	Dissolving HFMNs	directional barbs	DLP	Long-term TDD	[144]
*Drosophila* tarsal pads	Dual-suction-cup negative pressure adsorption	Dissolvable composite HFMNs	Conical MNs embedded in circular suction cup array	DLP	Bacterial stomatitis	[137]
Crocodile teeth	Enhanced anchoring via height gradient	Dissolving eutectic-gel MN	Customized shape, gradient height	DLP	Healing of infected wounds	[141]
Honeybee stingers	Mechanical interlocking	HMN	Two-layer, six 45° barbs per layer	PμSL	Chronic wound management	[134]
Backward-facing barbs	Sensing/triggered self-destruction	SMNs	Backward-facing barbs	LCD + double molding	Perioperative organ monitoring	[135]
*Drosera capensis*	AI-modeled shape memory coiling	SMNs	Curved, coiling needles	DLP-based 4D printing	Diabetic wound healing	[139]
Cactus spines/Wheat awns	Laplace pressure/hybrid harvesting	SMNs	Spine-grooved conical needles	PμSL	Freshwater-electricity cogeneration	[145]

**Fig. 5.**
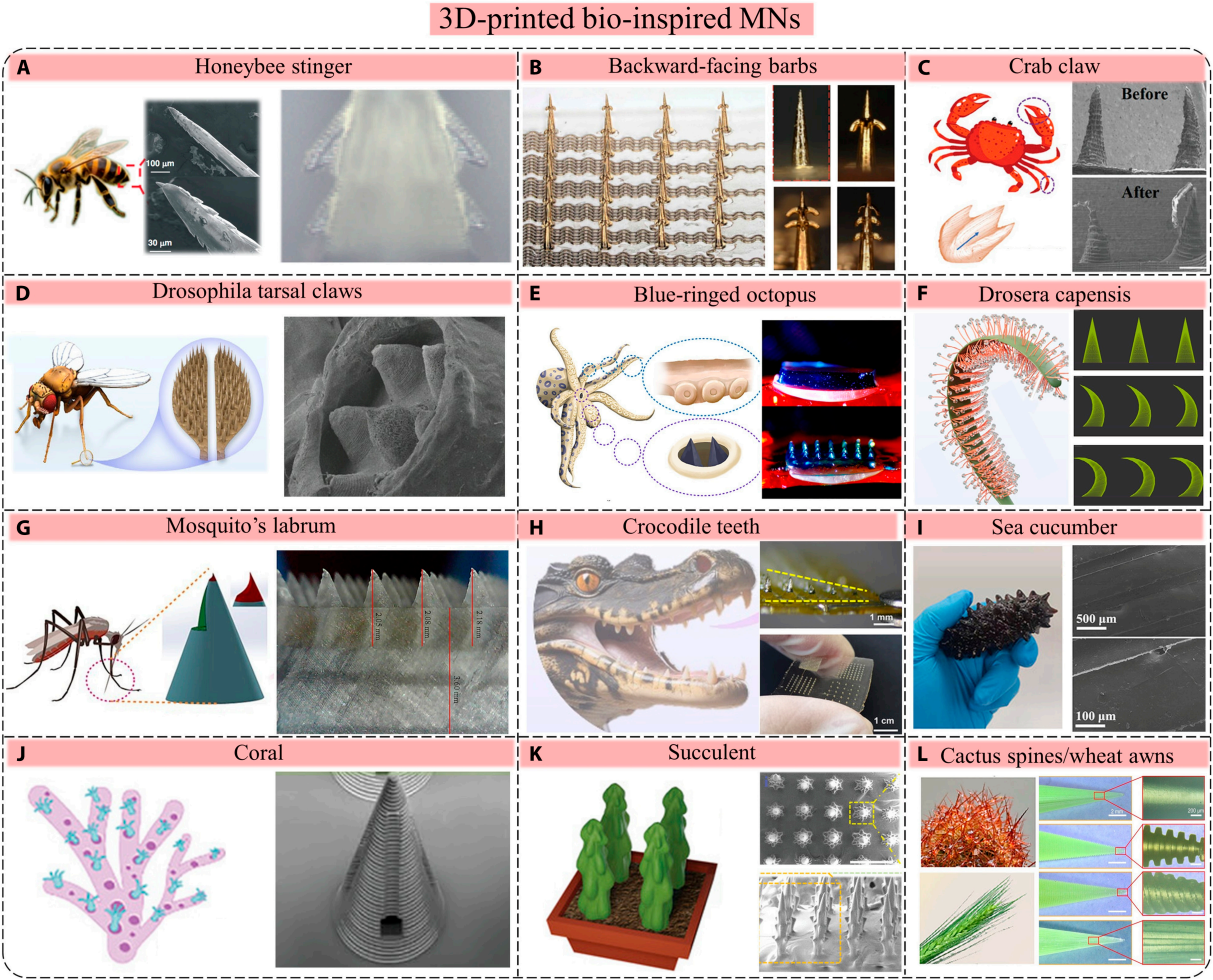
Bioinspired design strategies and 3D printing-based fabrication paradigms for multifunctional BMNs. (A) Honeybee stinger. Reproduced with permission from [134]. Copyright 2026, Springer Nature. (B) Backward-facing barbs. Reproduced with permission from [135]. Copyright 2026, Springer Nature. (C) Crab claw. Reproduced with permission from [136]. Copyright 2024, Wiley-VCH. (D) *Drosophila* tarsal claws. Reprinted from [137] under a CC BY 4.0 license. (E) Blue-ringed octopus. Reproduced with permission from [138]. Copyright 2023, American Association for the Advancement of Science. (F) *Drosera capensis*. Reproduced with permission from [139]. Copyright 2026, Wiley-VCH. (G) Mosquito’s labrum. Reproduced with permission from [140]. Copyright 2024, Elsevier. (H) Crocodile teeth. Reproduced with permission from [141]. Copyright 2025, Wiley-VCH. (I) Sea cucumber. Reproduced with permission from [142]. Copyright 2024, American Chemical Society. (J) Coral. Reproduced with permission from [143]. Copyright 2024, Wiley-VCH. (K) Succulent. Reproduced with permission from [144]. Copyright 2024, Wiley-VCH. (L) Aactus spines/Wheat awns. Reproduced with permission from [145]. Copyright 2026, Wiley-VCH.

The flexibility of 3D printing is first demonstrated in the enhancement of interfacial adhesion and dynamic fixation. To improve stable application on moist or mobile tissues, researchers utilized PμSL to fabricate monolithic honeybee stinger-inspired MNs (Fig. 5A) [134]. These structures feature an optimized double-layered design with 45° barbs to achieve robust mechanical interlocking with wound dressings. This biomimetic geometry balances penetration efficiency with anchorage strength, ensuring long-term stability for continuous biosensing and on-demand drug delivery. To extend this stability from the skin surface to internal environments, bioresorbable MN arrays with backward-facing barbs have been engineered for conformal and stable interfacing with deep organs, enabling long-term, spatially mapped electrochemical monitoring of organ health during the perioperative period (Fig. 5B) [135]. Furthermore, by synergizing crab-claw grasping, shark-skin micro-grooves, and kirigami-inspired deformation mechanisms, deformable DNA hydrogel MN arrays achieved superior conformability on irregular skin surfaces (Fig. 5C) [136]. This strategy also extends to *Drosophila* tarsal claw-inspired composite patches, which utilize biomimetic suction cups to overcome detachment challenges in high-humidity mucosal environments (Fig. 5D) [137]. For active wet adhesion, blue-ringed octopus-inspired systems utilized 3D-printed molds to integrate negative pressure suction with tannic acid-mediated chemical bonding, embodying an integrated “structure–material–function” approach (Fig. 5E) [138]. Building on these dynamic mechanisms, artificial intelligence (AI)-guided 4D printing has facilitated the creation of carnivorous plant-inspired MNs that actively curl and grasp tissue in response to physiological temperatures, offering a self-driven solution for accelerated wound closure through bioinspired movement (Fig. 5F) [139]. Furthermore, 3D printing effectively circumvents the technical bottleneck of channel clogging; notably, mosquito labrum-inspired HMNs were fabricated with patented internal pathways to ensure unobstructed drug delivery (Fig. 5G) [140].

Advancements in manufacturing resolution have further enabled precision internal topologies and functional zonation. This precision facilitates functional partitioning via gradient designs; for instance, crocodile tooth-inspired nonuniform arrays were developed to balance deep anchoring with shallow controlled release by modulating needle heights (Fig. 5H) [141]. In the field of bioelectronic interfaces, sea cucumber podia-inspired conductive nerve conduits were designed to generate piezo-responsive electrical signals, successfully guiding Schwann cell migration for peripheral nerve regeneration (Fig. 5I) [142]. When confronting more challenging biological environments, 3D printing facilitates multi-dimensional and multi-mechanism integration. Coral-inspired biomimetic patches (HepMi-PCL) integrated a rigid PCL shell with an intelligent heparin hydrogel core, utilizing siphoning effects for visual infection monitoring via colorimetric changes (Fig. 5J) [143]. Additionally, succulent-inspired humidity-responsive systems leveraged the differential swelling ofpoly(ethylene glycol) diacrylate (PEGDA) bases and moisture-sensitive hyaluronic acid methacryloyl (HAMA) tips to form reversible microhooks upon hydration, enabling self-locking and environment-triggered release (Fig. 5K) [144]. Notably, the versatility of 3D-printed biomimetic MNs now extends beyond biomedical interfaces to environmental resource harvesting; by mimicking the conical geometry and microgrooves of cactus spines and wheat awns, researchers developed an integrated trinity system capable of simultaneous fog collection and electricity cogeneration through spontaneous, directional water transport (Fig. 5L) [145].

In conclusion, 3D printing technology demonstrates an unparalleled capability in the fabrication of complex, nonstandard, and responsive multi-scale structures. Its highly flexible construction methodology not only enables the precise reconstruction of nature-inspired structural prototypes but also facilitates the integration of multifunctional modules, such as electrical conduction, sensing, and controlled release. This systemic design approach, which integrates structure, fabrication, and function, effectively overcomes the limitations of conventional MNs often characterized by monolithic structures and fragmented functionalities. Consequently, this advanced fabrication strategy establishes a new paradigm for the intelligent, personalized development of minimally invasive biomedical devices. Ultimately, these high-fidelity manufacturing capabilities and topology-driven engineering breakthroughs are not merely geometric milestones; they serve as the direct enabling foundation that dictates the platform’s functional performance when confronting complex biological barriers in vivo.

## Applications of 3D-Printed MNs in Biomedical Fields

The ability to create complex, nonplanar, and multi-material structures opens the door to a wide range of biomedical applications. Based on system complexity and functional mechanisms, these applications are categorized into 2 primary domains. Standalone MN patches capitalize on material-intrinsic and geometric innovations; by directly encoding therapeutic functionalities into the needle topologies (such as bioinspired barbs or stimuli-responsive matrices), they offer autonomous, cost-effective solutions optimized for localized barrier remodeling and decentralized patient self-care without the burden of external hardware. Conversely, integrated MN systems advance this biointerface baseline toward macro-system orchestration; by serving as micro-architectural hosts that embed flexible bioelectronics and programmable microfluidic networks, they achieve real-time physiological digitization and feedback-driven interventions for volatile metabolic environments. Consequently, this section systematically reviews the recent progress of 3D-printed MNs across these 2 functional domains.

### Standalone MN patches

Standalone MN patches represent the fundamental application of 3D printing in biomedicine, functioning without reliance on external electronics or power sources. By exploiting customizable geometries and responsive materials, these devices can overcome biological barriers to achieve targeted delivery or physical intervention. The following subsections detail their advancements across 3 dimensions: superficial wound and skin management, systemic metabolic and deep-tissue repair, and targeted tumor therapy and immune engineering.

#### Wound management and skin barrier remodeling

As the primary interface for MN application, the skin presents diverse pathological challenges ranging from open defects to inflammatory disorders. 3D printing enables the tailoring of MN structures to address these specific conditions. This subsection classifies these applications into 2 categories based on skin integrity: the repair of damaged skin and the regulation of intact skin.

#### Repair of Damaged Skin

In the management of damaged skin tissues, such as diabetic ulcers, deep-seated abscesses, and mucosal defects, conventional topical therapies are severely limited by their inability to penetrate bacterial biofilms and necrotic tissue, as well as their tendency to detach in moist, exudative environments. 3D-printed MNs offer a targeted solution to these challenges by leveraging their customizable structural advantages. Their sharp, high-aspect-ratio, or bioinspired geometric designs enable them to physically puncture dense biofilms and necrotic layers, delivering antimicrobial agents or angiogenesis-promoting factors directly to the deep infection core or ischemic tissue, thereby overcoming drug resistance and low deep-tissue bioavailability. Furthermore, 3D printing facilitates the fabrication of specialized interlocking structures that anchor the patch onto slippery, wet wound surfaces, ensuring sustained drug release and mechanical support necessary for accelerating wound closure.

**Fig. 6.**
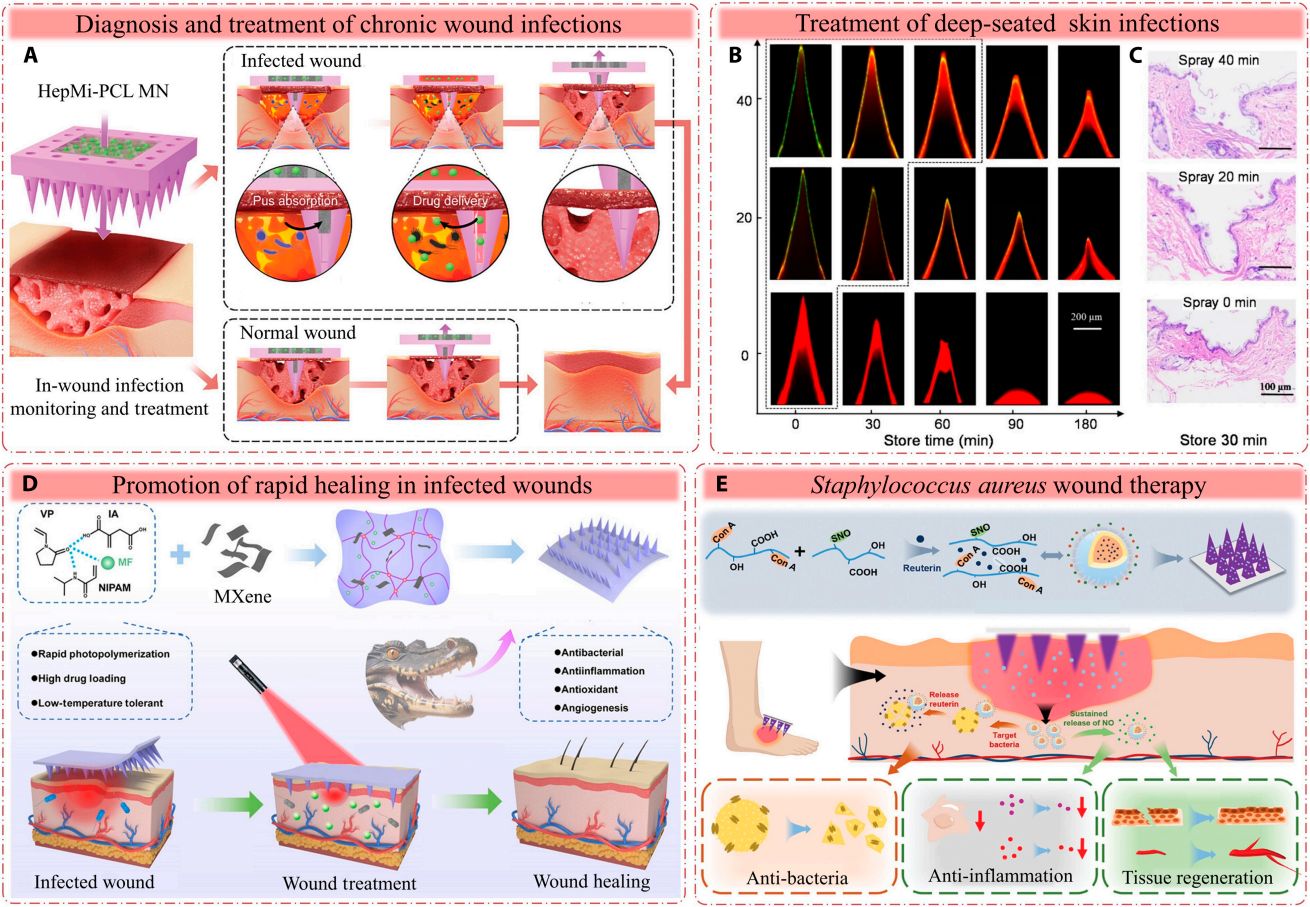
3D-printed MNs for infected wound management and deep abscess treatment. (A) PH-responsive HepMi-PCL MNs that autonomously release drugs in purulent, infected wounds and taper dosing to cessation upon resolution of infection. Reproduced with permission from [143]. Copyright 2024, Wiley-VCH. (B) Effect of spray-coating duration on MN morphological stability in high-humidity environments. (C) Hematoxylin and eosin (H&E)-stained skin sections from mice after different MN treatments. Reproduced with permission from [146]. Copyright 2024, Elsevier. (D) Schematic of NIR-responsive drug release and accelerated healing by MXene hydrogel MNs inspired by the gradient structure of crocodile teeth. Reproduced with permission from [141]. Copyright 2025, Wiley-VCH. (E) Targeted antibacterial action and NO-mediated anti-inflammatory/pro-regenerative synergy of RE@SA–Con A/SNO nanocarrier MNs. Reproduced with permission from [149]. Copyright 2025, Wiley-VCH.

Effective management of chronic infected wounds requires not only deep-tissue penetration but also real-time monitoring of the pathological state. The HepMi-PCL proposed by Liu et al. [143] demonstrated the integration of diagnostic and therapeutic functions, with its working mechanism shown in Fig. 6A. This MN system can penetrate the scab barrier. The internal lyophilized hydrogel becomes activated upon absorbing pus or exudate through the microporous structure, and the backing hydrogel rapidly turns red in the alkaline pus environment, enabling rapid visual diagnosis of infection. As the infection severity decreases and exudate reduces, the hydrogel gradually dries, correspondingly reducing or even terminating drug release, thereby achieving on-demand drug delivery and effectively preventing antibiotic misuse. In vitro experiments showed a bactericidal rate exceeding 95% against *Staphylococcus aureus* (MRSA) and *Escherichia coli*, and the wound healing speed in the treatment group increased by over 200%, demonstrating outstanding clinical translation potential. However, the high-humidity environment of infected wounds often compromises the structural integrity of MNs, limiting their efficacy in deep-tissue delivery. Addressing the challenges of low traditional transdermal delivery efficiency and bacterial drug resistance in deep skin infections, Zheng et al. [146] developed a dual-drug loaded CSMN system. This study combined 3D-printed polydimethylsiloxane (PDMS) molds with electrospraying techniques to construct a composite PB-PVP-MN@CUR-PLGA MN system. This system features a core composed of polyvinylpyrrolidone (PVP) loaded with the hydrophilic drug polymyxin B (PB), which is then coated with poly (lactic-co-glycolic acid) (PLGA) particles encapsulating the hydrophobic drug curcumin (CUR). As shown in Fig. 6B and C, the PLGA shell markedly enhanced the stability of the MNs in high-humidity environments, maintaining an effective penetration depth of 610 ± 82 μm even after 1 h of storage at 85% relative humidity. The dual-drug synergistic release achieved up to 99% reduction in MRSA biofilm activity in vitro and resulted in complete healing of a mouse subcutaneous abscess model within 12 d, demonstrating superior efficacy compared to traditional topical administration. This study provides a new strategy for infection treatment in high-humidity environments, although future work needs to optimize the adhesion of the PLGA coating to prevent drug particle shedding and validate its efficacy against resistant strains like methicillin-resistant MRSA.

Beyond chemical stability, ensuring secure mechanical fixation on dynamic skin surfaces is equally critical for consistent therapeutic outcomes. Inspired by the superior tissue-anchoring capability of crocodile teeth, Liu et al. [141] constructed a biomimetic gradient MN system (MF-MXene@MN) (Fig. 6D). This MN employs a combination of long and short needles, where the longer needles enhance tissue anchoring, while the shorter needles enable controlled release functionality. By integrating the photothermal conversion properties of MXene with the temperature/pH dual-responsive capability of the polymerizable deep eutectic solvent (PDES), the system achieves on-demand drug release upon near-infrared (NIR) light irradiation. The biomimetic gradient structure significantly improves the adhesive stability of the MN, preventing displacement or secondary damage. Experiments showed that, combined with NIR irradiation, the system accelerated the closure of infected wounds (healing rate reached 94.88% in 10 d) and promoted angiogenesis 3.2 times more than the control group, demonstratingsubstantially superior efficacy compared to traditional dressings and homogeneous MN structures. Despite improvements in stability and fixation, the dense bacterial biofilm barrier remains a formidable obstacle that often leads to treatment failure [147,148]. Targeting this challenge, Jin et al. [149] designed an MN patch loaded with engineered nitric oxide (NO) nanocarriers (Fig. 6E). This system employs alginate-based nanoparticles to co-load the antibacterial agent reuterin, the targeting molecule concanavalin A (Con A), and an NO donor. Con A specifically recognizes bacterial surface polysaccharides, enabling targeted sterilization, while the synergistic effect of reuterin and NO clears 78.13% of bacterial biofilm and sustains NO release for over 12 h, effectively down-regulating pro-inflammatory factors and promoting endothelial cell migration. In an infected mouse model, this MN system successfully penetrated the biofilm and achieved a 98.75% wound healing rate within 9 d, markedly promoting epithelial regeneration and collagen deposition. Although this system integrates antibacterial, anti-inflammatory, and pro-regenerative functions, issues such as the limited stability of Con A and the requirement for low-temperature, light-protected storage of the NO carriers still hinder its clinical translation. Future efforts are urgently needed to optimize the material’s stability and biocompatibility.

**Fig. 7.**
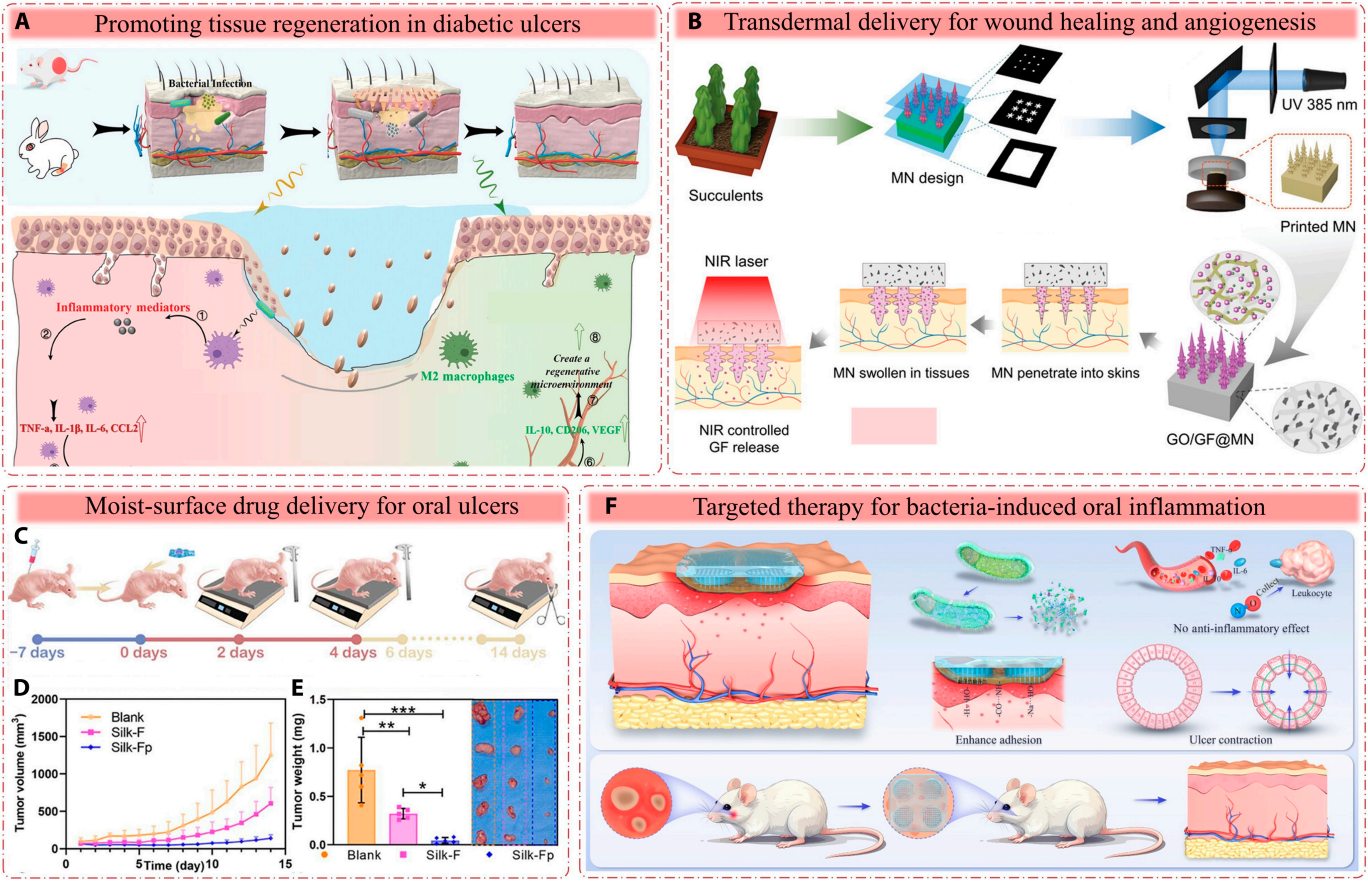
3D-printed MNs for ischemic wound healing and wet mucosal repair. (A) Schematic of P-DNA gel MNs for improving the healing of diabetic ulcer wound. Reproduced with permission from [136]. Copyright 2024, Wiley-VCH. (B) 3D-printed MN patch with succulent-inspired architectures enabling light-controlled sustained drug release. Reproduced with permission from [144]. Copyright 2024, Wiley-VCH. (C) Schematic of the treatment regimen for superficial tumors in nude mice. (D) Average tumor growth curves. (E) Post-treatment tumor photographs and weight statistics. Reproduced with permission from [138]. Copyright 2023, American Association for the Advancement of Science. (F) Working principle of the MN suction cup and its effect on ulcer healing in a rat burn model. Reprinted from [137] under a CC BY 4.0 license.

For chronic ischemic wounds, particularly diabetic ulcers, restoring the impaired microenvironment and re-establishing blood supply are fundamental challenges for tissue regeneration. In the treatment of diabetic ulcers, Zhou et al. [136] developed a polypeptide deoxyribonucleic acid hydrogel MN (P-DNA gel MN) patch loaded with extracellular vesicles extracted under hypoxia (H-EVs-hypoxia), applying it to a full-thickness infected skin defect animal model; the technical pathway is illustrated in Fig. 7A. Leveraging its biomimetic structure, this MN patch demonstrated excellent skin penetration capability and stable retention, effectively avoiding potential secondary wound damage caused by improper handling of traditional MNs. The loaded H-EVs-hypoxia modulated the wound microenvironment, promoting the proliferation and migration of fibroblasts and vascular endothelial cells, thereby accelerating re-epithelialization and granulation tissue formation. The treatment group achieved nearly complete healing within 22 d, exhibiting marked anti-inflammatory and antioxidant effects, effectively alleviating pathological pain, and promoting nerve and blood vessel regeneration. This study validates the multi-modal regulatory capacity of BMNs in complex wound environments, offering a new strategy for achieving high-quality tissue regeneration. Although comprehensive microenvironment modulation is effective, achieving precise spatiotemporal control over vascularization remains critical for reversing ischemia. Furthermore, in promoting vascular regeneration, Sun et al. [144] developed a composite MN system integrating thiolated heparin (Hep-SH) and graphene oxide (GO), achieving a light-controlled dual-modal drug release mechanism; its structure and drug release mechanism are illustrated in Fig. 7B. Using vascular endothelial growth factor (VEGF) as a model drug, the study leveraged electrostatic interactions between Hep-SH and the positively charged growth factor to prolong its release duration while introducing NIR light responsiveness via GO. In vitro experiments demonstrated that VEGF-loaded MNs could considerably promote the proliferation, migration, and tube formation of human umbilical vein endothelial cells (HUVECs). Specifically, under daily NIR irradiation, the GO/VEGF@MN + NIR group exhibited the most superior performance in cell migration and tube formation capability, validating that exogenous light can real-time enhance drug release efficiency and bioactivity. This offers a novel strategy for the spatiotemporally controlled release of growth factors during wound healing.

Unlike external skin wounds, mucosal injuries in wet environments pose unique difficulties due to continuous fluid secretion, which severely compromises patch adhesion and drug retention. Inspired by the sucker structure of the blue-ringed octopus, Zhu et al. [138] developed a BMN patch with strong wet adhesion and dual-mode drug release capabilities. In an oral ulcer model, MNs loaded with dexamethasone (DEX) effectively penetrated the mucus layer, achieving rapid absorption and sustained release, which substantially promoted healing and suppressed inflammation. In melanoma treatment, the 5-fluorouracil (5-FU)-loaded MNs utilized a combined strategy of active drug injection and sustained release, inhibiting tumor proliferation in the early stage and maintaining drug concentration later, ultimately restricting tumor volume to less than 5% of the control group and markedly inducing tumor cell apoptosis (Fig. 7C to E). This system demonstrated stable adhesion and drug release capability even in complex wet environments, providing an effective technical pathway for solving the challenge of drug delivery to moist tissues. Beyond passive adhesion and drug release, integrating active mechanical intervention offers a more dynamic strategy to accelerate mucosal healing. Unlike traditional hydrogels that rely on passive diffusion, bioinspired active systems can physically pull wound edges together, demonstrating substantial potential in managing complex oral diseases like bacterial infectious stomatitis (BIS) [150,151]. Inspired by the tarsal claw of *Drosophila*, Qin et al. [137] developed a BMN system integrated with a multifunctional hydrogel; its working principle and application effects in a rat BIS model are shown in Fig. 7F. This MN system utilizes a chitosan derivative as the antibacterial component, effectively inhibiting common oral pathogens like MRSA. Concurrently, the incorporation of the thermoresponsive polymer poly(N-isopropylacrylamide) (PNIPAM) enables contraction upon body temperature stimulation, actively pulling the wound edges to accelerate closure and facilitating the controlled release of an S-nitrosoglutathione (GSNO), thereby exerting anti-inflammatory and vasodilatory effects. By fusing biomimetic adhesion, mechanical contraction, and multiple biological activities, animal experiments showed complete closure of infectious ulcers within 6 d, highlighting its broad application prospects in treating complex oral diseases.

#### Regulation and Regeneration of Intact Skin

Extending beyond open wound management, 3D-printed MNs exhibit substantial therapeutic potential in treating dermatological disorders characterized by intact skin barriers that require precise modulation. The therapeutic efficacy of topical treatments for intact skin disorders, including psoriasis and hypertrophic scarring, is frequently compromised by the stratum corneum’s restriction on macromolecular transport. 3D-printed MNs address this challenge by creating reversible micro-channels that bypass the epidermal barrier, facilitating the targeted delivery of immunomodulators and bioactive agents to the dermis with minimal systemic exposure. Moreover, the geometric versatility of 3D printing enables the construction of patient-specific patches with conformal adhesion to irregular body surfaces, greatly amplifying therapeutic performance in aesthetic and regenerative applications.

**Fig. 8.**
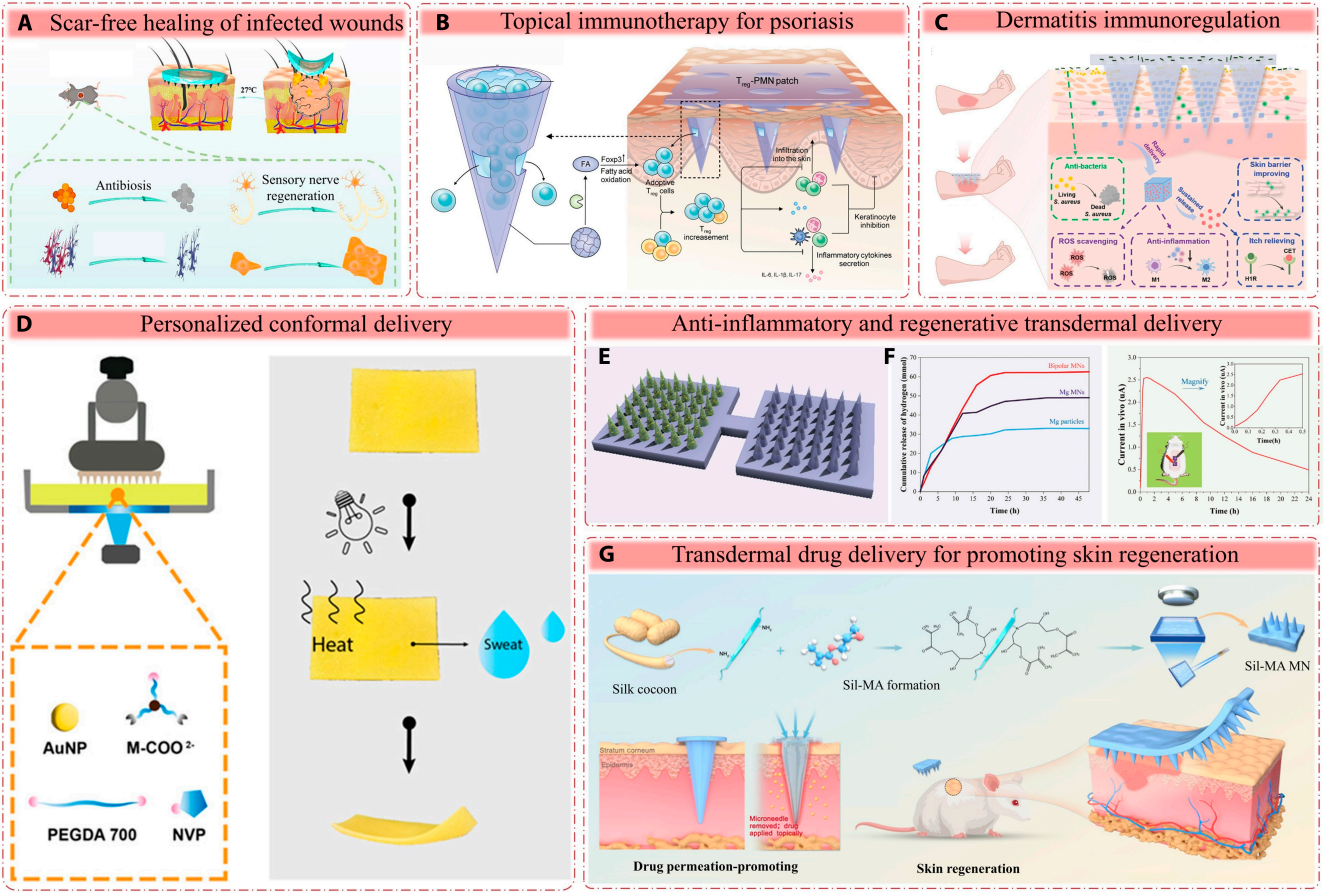
3D-printed MNs for scar management, immune-mediated therapy, and skin regeneration. (A) TPDM promoting healing and inhibiting scarring through sensory nerve regulation. Reproduced with permission from [157]. Copyright 2025, Wiley-VCH. (B) Mechanism of PMN patches for psoriasis therapy. Reprinted from [158] under a CC BY 4.0 license. (C) Mechanism of a bilayer Bs/CET@PB MN patch for AD treatment. Reproduced with permission from [159]. Copyright 2025, Wiley-VCH. (D) Materials composition and mechanism of an ion-responsive personalized transdermal patch. Reproduced with permission from [161]. Copyright 2024, American Chemical Society. (E) Schematic of the GCMN patch. (F) Schematic of hydrogen generation and microcurrent production. Reproduced with permission from [163]. Copyright 2025, Wiley-VCH. (G) Fabrication process of Sil-MA MNs and schematic of their application in skin regeneration. Reproduced with permission from [164]. Copyright 2025, American Chemical Society.

For skin tissues that have closed but exhibit pathological remodeling, such as hypertrophic scars, modulating the mechanical microenvironment is a novel therapeutic avenue. Existing therapies, such as surgical excision, pressure garments, and laser interventions, are generally limited by their invasiveness, marked side effects, and prolonged treatment duration [152–154]. Besides, the healing of infected wounds is often delayed by bacterial infection, chronic inflammation, and sensory nerve damage, and is prone to scarring, making treatment highly challenging [155,156]. Su et al. [157] developed a temperature-programmable deformable MN (TPDM) system, whose mechanism for promoting healing by modulating sensory nerve function is depicted in Fig. 8A. This MN can deform at elevated temperatures and revert to its initial shape at body temperature, effectively avoiding damage to normal tissues caused by heat treatment. The system utilizes gelatin methacryloyl (GelMA) as the matrix, integrating drug-loaded nanocapsules and a heterogeneous structured PANI@PDI with antibacterial functionality, thereby achieving a synergistic effect of antibacterial action, nerve repair, and anti-scarring. Experiments confirmed that the TPDM system achieved a 99.7% antibacterial rate against MRSA, promoted sensory nerve axon regeneration 2.71 times compared to the control group, and considerably inhibited fibroblast activity, thereby reducing scar formation. Although this system shows excellent performance in infection control and regeneration promotion, its broad-spectrum antibacterial capability, long-term stability, and biocompatibility still require further validation.

While structural remodeling targets physical defects, treating inflammatory dermatoses requires precise modulation of the immune microenvironment and skin barrier function. In 2023, Zhang et al. [158] proposed a PMN for enhanced delivery of regulatory T cells (Tregs) to treat psoriasis. This system utilizes an enzymatically degradable copolymer, poly(VP-co-MMA), as the matrix, providing both good mechanical strength and biodegradability. As shown in Fig. 8B, after the MNs penetrate the skin, Tregs migrate to the lesion site through internal channels, while the matrix releases propionate in the inflammatory environment to enhance Treg function, markedly increasing Foxp3 expression and boosting immunosuppressive capacity by 2 to 4 times. In a mouse psoriasis model, the PMN effectively alleviated inflammation and keratinocyte hyperplasia, demonstrating favorable therapeutic efficacy. Nonetheless, further optimizing the material degradation behavior and release kinetics is necessary to improve treatment precision and consistency. Besides, addressing the long-standing challenges of insufficient drug penetration and limited treatment options for atopic dermatitis, Zhang et al. [159] developed a bilayer MN patch (Bs/CET@PB MN), whose therapeutic mechanism is depicted in Fig. 8C. In this system, the needle tip layer is loaded with cetirizine–Prussian blue nanoparticles (CET@PB NPs), which scavenge reactive oxygen species (ROS), alleviate pruritus, and promote the expression of barrier proteins (such as FLG and LOR). The base layer incorporates *Bacillus subtilis* to improve the skin microbiome by competitively inhibiting MRSA. Animal experiments demonstrated that the patch possesses marked anti-inflammatory, antibacterial, and barrier repair functionalities. The drug delivery efficiency reached 87.5%, CET release was sustained for 12 d, and the viability of the bacteria exceeded 9 d. However, the bacterial delivery efficiency of this system was only 50.7%, and its applicability across different skin types and disease stages requires further investigation.

In the field of personalized TDD, achieving effective adhesion on curved skin surfaces remains a substantial challenge, as traditional planar patches often exhibit poor conformability [160]. Addressing this limitation, Zhou et al. [161] developed an ion-responsive MN patch based on photopolymerization 3D printing, with its material composition and mechanism of action illustrated in Fig. 8D. This system incorporates a shape memory polymer and an optimized photoinitiation system, enabling the controlled fabrication of high-precision MN arrays. The patch utilizes photothermal gold nanoparticles (AuNPs) to induce sweat secretion containing Na^+^, which triggers an ion exchange reaction causing the patch to bend and conform closely to the skin, therebynotably enhancing drug delivery efficiency. Furthermore, the AuNPs, modified with mercaptoundecanoic acid (MUA), confer the ability to retard drug release and enhance controlled release performance. Although this technology demonstrates promising potential for personalized adaptation, the residual photoinitiators and material biosafety require systematic evaluation, with future efforts needing to focus on biocompatibility and in vivo safety validation. Beyond physical adaptation, 3D-printed MNs offer innovative biofunctional strategies for reversing skin aging and promoting tissue regeneration. UV light-induced photoaging leads to the degradation of dermal collagen fibers, enhanced inflammatory responses, and wrinkle formation, with existing therapies often failing to achieve complete reversal [162]. Lin et al. [163] constructed a galvanic cell MN (GCMN) patch, whose structure and hydrogen generation mechanism are shown in Fig. 8E and F, respectively. This system integrates a magnesium (Mg) anode and a conductive cathode within a PLA/polyethylene glycol (PEG)/dopamine-functionalized polypyrrole (DA-PPy) substrate, utilizing tissue fluid as the electrolyte to trigger a magnesium–water reaction. This results in the continuous release of hydrogen and magnesium ions alongside microcurrent generation. Experiments demonstrated that the GCMN can scavenge ROS, promote cell migration, and substantially improve photoaging-induced wrinkles by inducing macrophage polarization toward the M_2_ phenotype. In addition to anti-aging applications, high-precision manufacturing is equally vital for functional tissue regeneration applications such as hair growth. To further enhance the manufacturing precision and mechanical properties of SF-based MNs, Tong et al. [164] developed a methacrylated SF (Sil-MA) bioink and successfully fabricated high-precision MN arrays by combining it with DLP printing technology. By optimizing the ink formulation and printing parameters, the resulting MNs exhibited excellent geometric precision (tip diameter ~40 μm), mechanical strength (2.9 N/needle), and biocompatibility (cell viability > 97%). As shown in Fig. 8G, the Sil-MA MNs markedly enhanced the transdermal delivery efficiency of various drugs (such as methotrexate, minoxidil, and ferulic acid) and promoted keratinocyte proliferation and skin regeneration through mechanical stimulation. Although this system demonstrates outstanding structural and performance characteristics, its long-term biosafety and capacity for complex drug delivery require further validation to accelerate its clinical translation process.

#### Metabolic management and deep-tissue repair

Extending beyond localized cutaneous therapy, 3D-printed MNs are emerging as sophisticated interfaces for systemic metabolic regulation and deep-tissue regeneration. The effective management of these diseases necessitates long-term monitoring and sustained drug delivery, posing substantial challenges for existing healthcare models. While traditional management of chronic metabolic diseases is often hindered by low bioavailability and poor compliance, MNs effectively access the dermal microcirculation to enable systemic delivery of labile biological agents. Furthermore, addressing the mechanical challenge of stabilizing devices on dynamic, wet internal organs, such as the beating heart or damaged nerves, 3D printing enables the fabrication of bioinspired anchoring structures that achieve robust mechanical interlocking without sutures. This section explores how these structural innovations empower MNs to transcend superficial barriers, enabling intelligent metabolic control and precise deep-organ intervention [142,165–167].

**Fig. 9.**
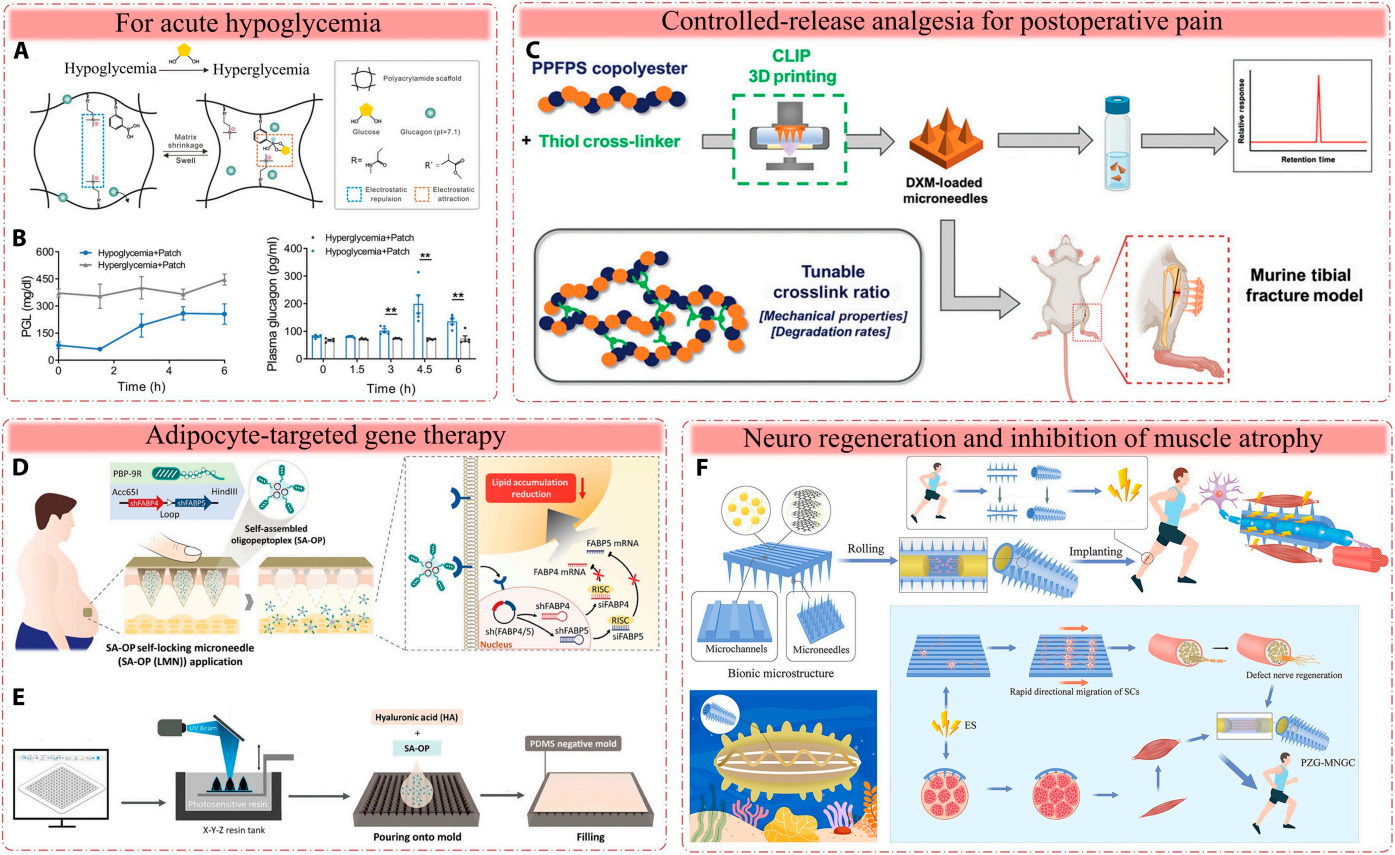
3D-printed MNs for systemic metabolic regulation and deep-tissue repair. (A) Schematic of the mechanism of a GRS glucagon delivery system. (B) Effects of the patch under different glycemic states and insulin treatments in diabetic mouse models. Reprinted from [165] under a CC BY 4.0 license. (C) Mechanism and application of the LMN patch. Reproduced with permission from [166]. Copyright 2024, Wiley-VCH. (D) Fabrication process of the LMN patch. (E) Fabrication workflow of MAPs and schematic representations of in vitro drug loading and in vivo therapeutic efficacy evaluation. Reproduced with permission from [167]. Copyright 2024, Wiley-VCH. (F) Schematic of ZnO NPs and rGO (PZG)-MNGCs suppressing muscle atrophy and promoting peripheral nerve regeneration under electrical stimulation. Reproduced with permission from [142]. Copyright 2024, American Chemical Society.

For metabolic diseases requiring precise dosage control, smart responsive DDSs represent a paradigm shift and have emerged as a major research hotspot, particularly for the dynamic management of conditions like diabetes [168]. Traditional blood glucose control methods rely on patient-initiated monitoring and manual intervention, a process that carries risks of delayed response and improper dosage control. To address the challenge of emergency intervention for hypoglycemia, Wang et al. [165] developed a glucose-responsive glucagon MN patch. This system employed a shrinking microfabrication strategy, where dehydration induced the contraction of large-sized hydrogel MNs, effectively circumventing the need for high-precision equipment and complex material purification required in traditional MN fabrication. Crucially, the matrix material, a cationic polyacrylamide (PAMS)/phenylboronic acid copolymer, possesses charge reversal capabilities, enabling responsiveness to blood glucose fluctuations. This glucose-responsive shrinking (GRS) system swells in the matrix under hypoglycemic conditions to accelerate glucagon release, while it contracts under hyperglycemic conditions to suppress release, thereby establishing a closed-loop feedback control mechanism (Fig. 9A). As shown in Fig. 9B, the system achieved autonomous and dynamic blood glucose regulation in a diabetic mouse model, providing a novel paradigm for smart MN design and opening avenues for developing delivery platforms responsive to other biomarkers. In addition to smart responsiveness, expanding the payload capability to include sustained release formulations and complex biologics is essential for effective systemic therapy. 3D-printed MN technology has facilitated a paradigm shift from the immediate release of traditional small molecules to the programmable sustained delivery of drugs and the administration of complex therapeutics, including gene therapy vectors [166]. For example, Bahnick et al. [166] utilized CLIP technology to fabricate biodegradable MN arrays for the sustained delivery of DEX for postoperative pain management. Figure 9C shows the fabrication process and drug release evaluation results. The system allows programmable control over the MN’s mechanical strength, degradation rate, and drug release kinetics by adjusting the stoichiometry of the photo-curable resin. Pharmacokinetic studies showed that the system provided local therapeutic efficacy comparable to intravenous injection whilegreatly reducing systemic drug exposure, highlighting its potential for localized precision therapy and side effect mitigation. Moving to the realm of gene therapy, Choi et al. [167] developed a dissolvable locking MN (LMN) for delivering self-assembled oligopeptide complexes (SA-OP) to treat obesity. The mechanism of action and fabrication process of this patch are shown in Fig. 9D and E. This system considerably enhanced the stability and storage performance of fragile nucleic acid materials by encapsulating them within the MNs. Simultaneously, the patch utilized a painless, transdermal, self-locking delivery method, effectively improving both administration efficiency and patient compliance. Experimental results showed that this MN system successfully achieved targeted gene silencing in a mouse model and substantially improved metabolic indicators such as insulin resistance and hepatic steatosis. In the domain of peripheral nerve injury repair, Hu et al. [142] proposed a system integrating a piezoelectric MN with nerve guidance conduits (MNGCs), achieving synergistic therapy combining electrical stimulation and physical guidance. Following implantation, the MN tips anchor into the target muscle. Leveraging the piezoelectric effect generated by daily activities, the system continuously releases microcurrents to alleviate muscle atrophy caused by denervation. Furthermore, the microchannel structure within the conduit effectively guides nerve axon regeneration (Fig. 9F). This research fully demonstrates the unique advantages of 3D printing in constructing complex biomimetic structures and integrating treatment with medical devices, offering a new pathway to address mechanical and functional impairments in specific clinical settings.

#### Tumor therapy and immune engineering

Progressing from physiological tissue repair to the modulation of complex pathological microenvironments, 3D-printed MNs provide a versatile platform for oncology and immunology. Unlike the structural restoration required for wounds or organs, tumor therapy faces the dual hurdles of penetrating dense stromal barriers and overcoming immunosuppression. 3D-printed MN technology addresses these challenges by enabling the precise loading of complex biologics within sophisticated architectures, encompassing areas such as tumor microenvironment modulation, controlled release of antitumor agents, and vaccine delivery. By directly delivering chemotherapeutics or immunomodulators to target tissues, this platform not only markedly enhances local therapeutic efficacy but also effectively minimizes the systemic toxicity and side effects commonly associated with traditional systemic administration.

**Fig. 10.**
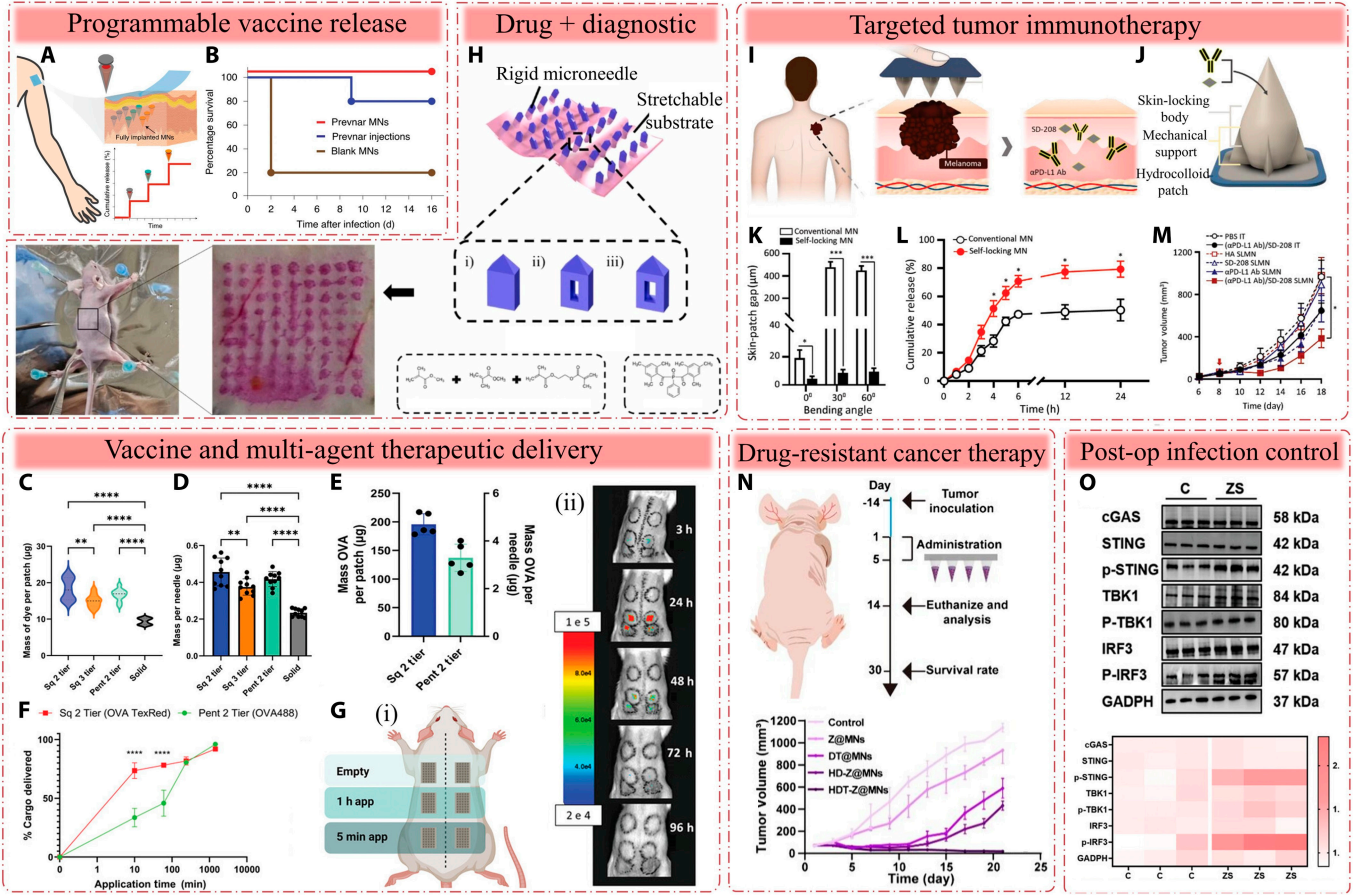
3D-printed MNs for precision tumor therapy and vaccine delivery. (A) Schematic of a PLGA core–shell transdermal MN patch. (B) Survival rates of experimental mice with or without pneumococcal vaccine (Prevnar-13). Reproduced with permission from [169]. Copyright 2021, Springer Nature. (C) Quantitative comparison of solid-state drug loading capacity for different MN structures. (D) Statistical analysis of drug loading. (E) Quantification of mRNA–LNP loading within MNs. (F) Release kinetics of dual fluorescently labeled proteins. (G) In vivo bioluminescence imaging comparisons. Reprinted from [170] under a CC BY 4.0 license. (H) Schematic diagram of ex vivo penetration tests on mouse skin and drug release experiments. Reproduced with permission from [70]. Copyright 2024, Elsevier. (I) Mechanism of self-locking DMN patches in melanoma immunotherapy. (J) MN array with integrated 4-wing mechanical supports for enhanced mechanical stability. (K) Effect of the MN–skin contact angle on interfacial gaps. (L) Comparison of skin permeation kinetics between self-locking DMNs and conventional MNs. (M) Tumor volume change in mice following treatment with αPD-L1 Ab/SD-208 self-locking DMNs. Reproduced with permission from [171]. Copyright 2023, Wiley-VCH. (N) Experimental design and tumor growth curves for combination therapy in animal models. Reproduced with permission from [172]. Copyright 2025, American Chemical Society. (O) Zinc–manganese sulfide/sparfloxacin (ZMS/SP) MNs synergistically activate the cGAS–STING signaling pathway. Reprinted from [173] under a CC BY 4.0 license.

3D-printed MNs provide a programmable platform for vaccine delivery that enhances immune activation while resolving the compliance challenges of conventional multi-dose regimens. For instance, to overcome the limitations of multiple injections required by conventional vaccines, Tran et al. [169] developed a transdermal MN patch with a core–shell structure (Fig. 10A). This system utilizes PVP as the carrier to form the MN core, which encapsulates vaccine components, while the shell is composed of PLGA with different molecular weights. By modulating the degradation kinetics of PLGA, a programmable, pulsatile release profile spanning from several days to 48 d was achieved. Furthermore, a single application of this MN patch induced an immune response comparable to multiple subcutaneous injections and provided 100% protection to rats against lethal infection (Fig. 10B). This research effectively broke through the bottleneck of traditional MNs offering only immediate or simple sustained release, avoiding the issue of immune tolerance caused by continuous antigen exposure. With the rise of nucleic acid vaccines, increasing loading capacity and maintaining the structural integrity of complex biologics become paramount for next-generation immunization. To achieve high-efficiency delivery of various biological agents, Rajesh et al. [170] developed lattice-structured MN array patches (L-MAPs) based on CLIP 3D printing technology. Through parametric design and computational modeling, this system constructed 3 distinct MN geometries with enhanced mechanical strength. Furthermore, this platform demonstrated a capacity for loading solid small-molecule drugs more than twice that of traditional MNs (Fig. 10C and D) and could load up to 850 ng/patch of mRNA-loaded lipid nanoparticles (mRNA-LNPs) (Fig. 10E). In a rat model, L-MAPs successfully delivered both protein antigens and mRNA (Fig. 10F and G), validating their broad applicability for the transdermal delivery of vaccines and various biologics.

Beyond preventive vaccination, effective eradication of established solid tumors requires structural capabilities to penetrate dense tumor stroma and maintain prolonged retention on dynamic skin surfaces. However, the poor conformability of traditional rigid MNs on dynamic skin surfaces and their propensity to fracture still limit their clinical application [160]. To overcome this challenge, Che Ab Rahman et al. [70] have developed a novel UV-curable PMMA resin system, addressing the limited availability of biocompatible materials suitable for VPP 3D printing. By introducing the crosslinker ethylene glycol dimethacrylate to enhance mechanical properties and employing the low-cytotoxicity photoinitiator BAPO, the biosafety of the material was greatly improved. As shown in Fig. 10H, the resulting MNs featured a side-opening hollow structure, which markedly enhanced drug loading efficiency. In the context of melanoma immunotherapy, Joo et al. [171] utilized PμSL technology to fabricate a dissolvable self-locking DMN patch (Fig. 10I). Its unique wide-body interlocking structure, along with mechanical support wings (Fig. 10J), considerably increased the success rate of skin penetration to 93.5% and improved the patch’s adhesion performance on dynamic skin surfaces (Fig. 10K). By optimizing drug distribution within the MNs, the transdermal release efficiency of the system was improved by 58% (Fig. 10L). In a melanoma mouse model, the patch co-delivered a transforming growth factor-β (TGF-β) inhibitor and an anti-PD-L1 antibody (αPD-L1 Ab), resulting in a marked inhibition of tumor growth (tumor volume inhibition rate up to 60%). The therapeutic efficacy was clearly superior to traditional intratumoral injection (Fig. 10M).

Beyond surface retention, overcoming multidrug resistance in deep-seated tumors requires precise subcellular targeting strategies. For the treatment of doxorubicin (DOX)-resistant breast cancer, Hao et al. [172] developed a mitochondria-targeting MN system (HDT-Z@MNs). This system utilized hollow nanoparticles as carriers, encapsulating DOX covalently coupled with the mitochondria-targeting molecule triphenylphosphonium. Following local application of the MN patch, the nanoparticles dissociate within the tumor microenvironment, driving drug accumulation within the mitochondria of tumor cells. The system synergistically activates both apoptosis and ferroptosis pathways by inducing a burst of ROS, glutathione (GSH) depletion, and glutathione peroxidase 4 (GPX4) inhibition. In animal experiments, a single administration inhibited tumor growth by 14-fold and substantially prolonged mouse survival to 48 d without observed systemic toxicity (Fig. 10N). Furthermore, preventing postoperative recurrence and metastasis remains a critical clinical challenge, necessitating systems that can activate systemic immunity. For the management of postoperative infection and metastasis, Chu et al. [173] developed an implantable HA MN system for triple-negative breast cancer that co-delivers the antibacterial drugSP and zinc–manganese sulfide nanoparticles. This MN system not only possesses antibacterial and anti-biofilm formation capabilities but also induces immunogenic cell death through the release of ROS and hydrogen sulfide (H_2_S) gas, thereby activating the cGAS-STING innate immune pathway (Fig. 10O). This system effectively inhibited primary tumor growth and markedly reduced lung metastasis, providing a safe and efficient therapeutic strategy for preventing and treating post-operative complications.

In summary, 3D-printed standalone MN patches exhibit broad applicability, progressing from superficial skin barrier remodeling and wound management to systemic metabolic regulation and tumor immune engineering. Current research focuses on overcoming critical limitations, including physiological barrier penetration, mechanical fixation on dynamic tissues, and the stabilization of complex bioactive molecules. To facilitate a comparative analysis, Table 4 systematically summarizes the core characteristics of these systems, covering material composition, printing techniques, structural designs, and key performance metrics.

**Table 4.** A comparative analysis of 3D-printed standalone MN patches for biomedical applications

Types	3D printing method	MN type	MN structure	MN material	Therapeutic agents	Advantages	Application	Ref.
Wound management and skin barrier remodeling (repair of damaged skin)	PμSL	Thermosensitive HFMNs	Conical array, H 500 μm	SilMA, F127DA, PNIPAM	DEX, 5-FU	Active injection	Wet tissue delivery	[138]
	3D-printed mold	Deformable HFMNs	H ~2.5 mm	Polypeptide-DNA hydrogel	EVs	Enhanced conformity	Diabetic ulcer	[136]
	DLP	PMNs	H 1,000 μm, porous	PCLMA, Hep-HA	MIN, Heparin	Theranostic, on-demand	Chronic wound infection	[143]
	PμSL	DMNs	Conical, H 790 μm, D_b 382 μm	PVP, PLGA	PB, CUR	Synergistic antibacterial	Deep skin infection	[146]
	DLP	DMNs	H 2.5 mm, barbed	PEGDA, PEGDA/HAMA	RhB, BSA, VEGF	Humidity-responsive adhesion	Long-term delivery	[144]
	DLP	Eutectogels DMNs	Personalized gradient H	PDES	MF	Intelligent release	Wound healing	[141]
	DLP	Composite DMNs	Conical, H 700 μm, D_b 250 μm	NACHG, PVA	GSNO	Wet adhesion, thermoresponsive	Bacterial stomatitis	[137]
	PμSL	DMNs	10 × 10 array, H 850 μm, D_b 400 μm	PVA, Sucrose	Reuterin, NO	Sustained release	Chronic wound therapy	[149]
Wound management and skin barrier remodeling (regulation and regeneration of intact skin)	SLA	HMNs	H 1,100 μm, 4 channels	Poly (VP-co-MMA)	Treg cells, Fatty acids	Localized targeting	Psoriasis	[158]
	DLP	SMNs	conical, H 800 μm, D_b 400 μm	PEGDA 700, MCOO-Ca, MUA-AuNPs	RhB	Photothermal release	Personalized delivery	[161]
	DLP	Bilayer SMNs	Conical, H 680.71 μm	GelMA, PANI@PDI	NGF, Verteporfin	Programmable deformation	Scarless healing	[157]
	PμSL	DMNs	Conical, H 850 μm, D_b 400 μm	PB NPs, polymer	Cetirizine, *B. subtilis*	ROS scavenging	AD	[159]
	SLA	SMNs	11 × 11 array, H 600 μm	PLA, PEG, DA-PPy, Mg	H_2_, microcurrent, Mg^2+^	Self-powered, synergistic	Skin repair	[163]
	DLP	SMNs	Conical, H 1,450 μm, D_b 1,000 μm	Sil-MA	MTX, minoxidil, ferulic acid	High precision	Skin regeneration	[164]
Metabolic management and deep-tissue repair	SLA	DMNs	Cone/pyramid, D_b 0.6 mm, H 0.8 mm	Acrylamide-MAETAC-APBA copolymer	Glucagon	RT-stable, washable	Acute hypoglycemia	[165]
	PμSL	SMNs	H 800 μm, D_b ~600 μm	PCL/rGO/ZnO (PZG)	/	Self-powered	Nerve repair	[142]
	PμSL	DMNs	Self-locking conical, H ~600μm	HA	SA-OP	Painless, stable	Obesity therapy	[167]
	CLIP	DMNs	8 × 8 array, H 1-2 mm	PPFPS, Thiol-ene	DEX	Controlled release, biodegradable	Post-op pain	[166]
Tumor therapy and immune engineering	TPP	CSMNs	H 600 μm, PVP core	PLGA, PVP, PLA	Prevnar-13, OVA	Single-injection mimic	Programmable vaccine	[169]
	PμSL	DMNs	H 700 μm, Support wings	HA	SD-208, αPD-L1 Ab	Enhanced adhesion	Melanoma immunotherapy	[171]
	DLP	SMNs with holes	0.25 × 0.5 × 1 mm, side holes	PMMA-based resin	RhB	High loading	Drug delivery	[70]
	CLIP	HMNs	H 1,200 μm, lattice	KeySplint hard resin	mRNA-LNPs, OVA	Tunable release	Vaccine delivery	[170]
	PμSL	DMNs	H 850 μm, D_b 400 μm	PVA/sucrose, PS	DOX-TPP	Synergistic death	Resistant breast cancer	[172]
	PμSL	DMNs	Conical, H ~600 μm, D_b ~200 μm	HA, ZnMnS NPs	SP, Zn^2+^, Mn^2+^	Antibacterial, immunoactivation	Post-op breast cancer	[173]

H, height; D_b, base diameter; BSA, bovine serum albumin; DOX-TPP, mitochondria-targeted doxorubicin-TPP; MF, mangiferin; MIN, minocycline hydrochloride; MTX, methotrexate; NGF, nerve growth factor; OVA, ovalbumin; RhB, rhodamine B; SD-208, TGF-β inhibitor; F127DA, polyether F127 diacrylate; Hep-HA, heparin-hyaluronic acid hydrogel; MAETAC, 3-[(methacryloylamino)propyl]trimethylammonium chloride; MCOO-Ca, calcium crosslinker; MUA-AuNPs, 11-mercaptoundecanoic acid functionalized gold nanoparticles; PANI@PDI, a composite material; PCLMA, methacrylated polycaprolactone; PP, polypropylene; PPFPS, poly [propylene fumarate-co-poly (sebacoyl fumarate)]; PS, polystyrene; PZG, PCL/rGO/ZnO NPs composite; rGO, reduced graphene oxide; ZnMnS NPs, ZnMnS nanoparticles

### Integrated MN systems

Tracing the progression from standalone functional matrices to system-level integration, this section delineates the convergence of MNs with advanced bioelectronics, microfluidics, and intelligent control architectures. This transition represents a profound paradigm shift driven by the imperative to transcend the spatiotemporal constraints of conventional personalized medicine. Standalone structures, despite their refined mechanical penetrability and geometric optimization, inherently operate within an open-loop, passive configuration, rendering them incapable of modulating their kinetic output or microfluidic transport profiles in response to nonlinear, real-time metabolic fluctuations. System-level integration radically addresses this vulnerability by orchestrating heterogeneous operational domains into a structurally unified and functionally coherent theranostic platform. By establishing an unbroken, bidirectional informational loop between in situ diagnostic networks and responsive actuation elements, integrated MN platforms evolve from descriptive mass-transport conduits into autonomous, feedback-driven medical devices. This synergistic coupling effectively eliminates intrinsic operational latencies and circumvents physical transport barriers, thereby defining the technological frontier of advanced precision healthcare. To systematically evaluate these breakthroughs, we categorize these developments into 2 interdependent functional domains: MN-enabled bioelectronic and microfluidic diagnostics for high-fidelity sensing, and MN-enabled field-assisted and feedback interventions for active therapeutic action

#### MN-enabled bioelectronic and microfluidic diagnosis

This category highlights the frontier of MN-enabled diagnostic systems, which synergize bioelectronic sensing interfaces with microfluidic analytical capabilities to achieve the high-fidelity digitization of multidimensional physiological signals. By serving as minimally invasive bridges, these systems facilitate the in situ acquisition of electrophysiological and physical data via direct electronic interfaces while simultaneously enabling the precise quantification of biochemical analytes through efficient biofluid extraction and microfluidic analysis. This convergence of electronic and fluidic modalities provides essential technological support for point-of-care testing (POCT), continuous dynamic monitoring, and the early identification of pathological states.

##### Bioelectronic Sensing

**Fig. 11.**
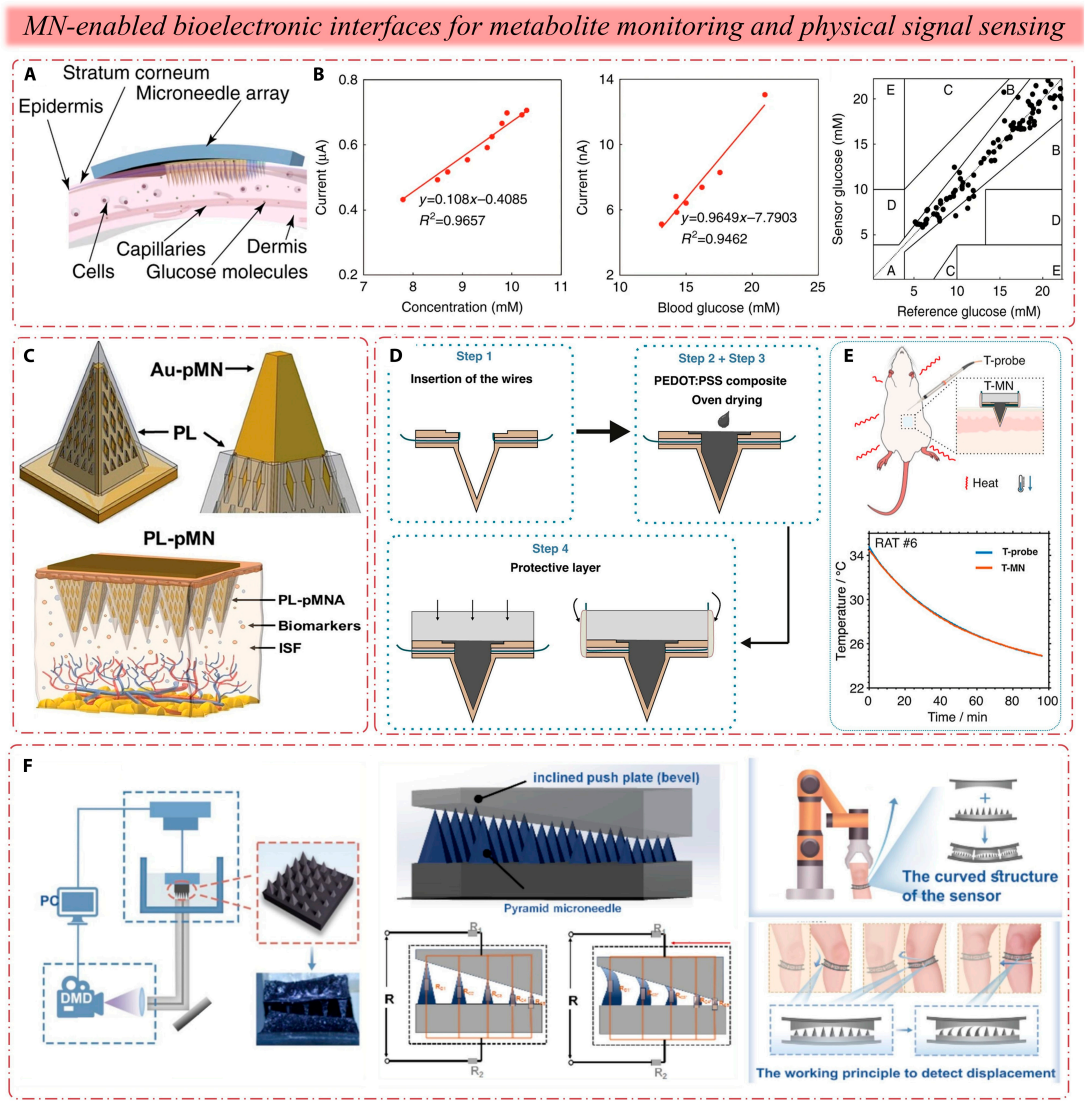
MN-enabled bioelectronic interfaces for metabolite monitoring and physical signal sensing. (A) Schematic of the integrated MN arrays penetrating the dermis and accessing ISF. (B) In vivo clinical accuracy evaluation via Clarke error grid analysis. Reprinted from [174] under a CC BY 4.0 license. (C) Schematic of the multifunctional PL-pMNA-based biosensing platform. Reproduced with permission from [175]. Copyright 2024, Wiley-VCH. (D) Fabrication process of T-MNs. (E) Deviation analysis between intradermal temperatures measured by T-MNs and commercial optical probes in vivo. Reprinted from [176] under a CC BY 4.0 license. (F) Sensing mechanism and application of 3D-printed PAMS hydrogel MNs for detecting knee joint micro-motion. Reproduced with permission from [178]. Copyright 2025, Elsevier.

For continuous metabolite monitoring, establishing a stable and sensitive electrochemical interface directly within the ISF is a prerequisite for clinical accuracy. In 2021, Liu et al. [174] reported an integrated 3D-printed MN biosensor for the continuous monitoring of glucose levels in the subcutaneous ISF of mice (Fig. 11A). This sensor integrated a PB-modified gold working electrode and an Ag/AgCl counter/reference electrode onto a single MN chip, utilizing immobilized glucose oxidase to catalyze the reaction of glucose in the ISF, thereby generating an electrical signal. The sensor exhibited a wide linear detection range of up to 24 mM in vitro. Furthermore, in a 7-d in vivo experiment, the monitoring results showed a high correlation with those from a commercial glucometer (*R*^2^ > 0.94), with all data points falling within the clinically acceptable zones of the Clarke error grid analysis (Fig. 11B). Although the sensor demonstrated excellent monitoring performance, its biocompatibility and adaptability to varying skin thickness and bioimpedance require further optimization, such as enhancing biosafety with a Nafion coating, to accelerate its clinical translation. However, the fabrication of complex 3D structures and the integration of protective modules remain major technical bottlenecks for the stability of such sensors. Addressing this, Dervisevic et al. [175] proposed a multifunctional PL-pMNA combining 3D printing and soft lithography techniques (Fig. 11C). This platform consists of gold-coated polymer MNs (Au-pMNA) and a polymer lattice (PL) protective film featuring a diamond micropore array, with a 25- to 40-μm offset gap reserved between them to balance electrochemical activity and mechanical protection of the sensing layer. Ex vivo experiments showed that the signal attenuation rate remained below 5% even after 3 repeated insertions, demonstrating excellent operational stability. Nonetheless, the platform’s capability for parallel detection of multiple biomarkers and its long-term reliability in vivo still require further optimization.

Beyond biochemical analysis, the high-fidelity acquisition of physical physiological signals, such as temperature and motion, is equally critical for comprehensive health assessment. Wei et al. [176] utilized high-resolution DLP 3D printing technology to fabricate a hollow pyramid-shaped temperature-sensing MN sensor (T-MN), demonstrating substantial potential for physiological parameter monitoring (Fig. 11D). The sensor is internally filled with a poly(3,4-ethylenedioxythiophene):polystyrene sulfonate (PEDOT:PSS) conductive composite, and the needle tip diameter is less than 45 μm, ensuring painless penetration and high-precision thermal response. Experimental results indicated that the sensor achieved a sensitivity of −0.74% °C^−1^ within the 15 to 60 °C range, a temperature resolution of 0.2 °C, and repeatability and reproducibility errors of only 2%, with performance stability for up to 60 d. As shown in Fig. 11E, in in vivo rat experiments, the temperature measured by the T-MN deviated by only ±0.1 °C compared to a commercial optical probe. This highlights its application prospects for monitoring pathological states such as inflammation and infection, along with its potential function to provide temperature compensation for other biosensors. While rigid sensors excel in stability, traditional materials often lack the flexibility and biocompatibility required to perceive minute mechanical displacements in dynamic joints. Hydrogel materials, owing to their mechanical properties similar to human tissues and good conductivity, have become key candidate materials for next-generation flexible sensors [177]. Zhang et al. [178] developed a highly sensitive hydrogel MN sensor based on 3D printing technology for knee joint micro-motion monitoring. The working mechanism and application are shown in Fig. 11F. By optimizing a PAMS hydrogel formulation and incorporating MXene nanomaterials to enhance conductive properties, this sensor achieved precise detection of minute displacements at the 20-μm level, with a high gauge factor of 7.74. It successfully captured sliding displacements of 40 to 200 μm and joint torsion angles of 5° to 20° in a knee joint model, and it was able to identify abnormal mechanical behaviors in patients with flatfoot and osteoarthritis. Although the sensor demonstrates excellent performance, it currently faces limitations such as insufficient response to high-frequency distortion (>2 Hz), lack of integrated therapeutic functions, and a limited sample size. Future work should focus on developing multi-modal sensing mechanisms to enhance its applicability in complex physiological environments.

**Fig. 12.**
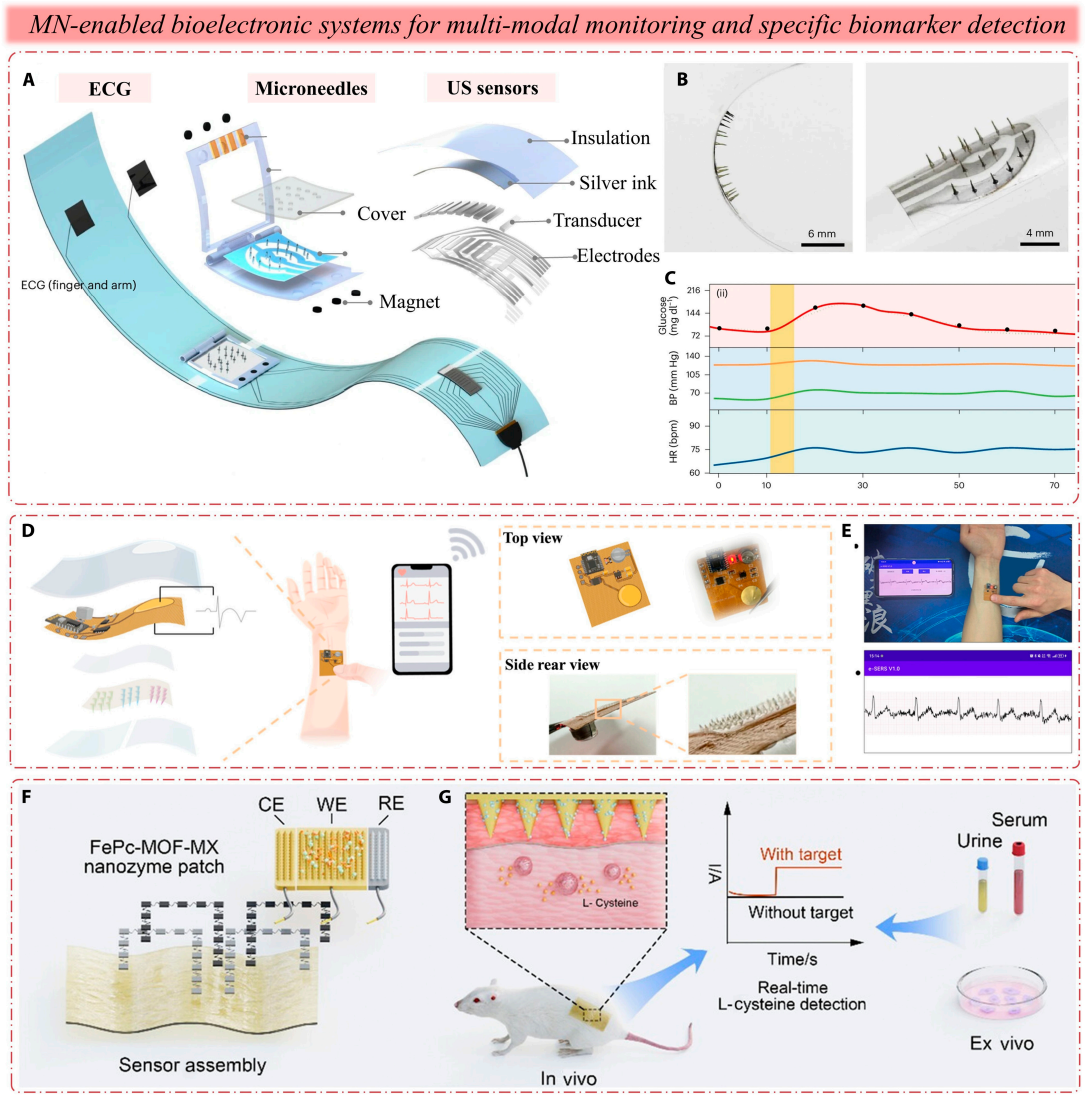
MN-enabled bioelectronic systems for multi-modal monitoring and specific biomarker detection. (A) Structural representation of the BLUE system. (B) Demonstration of MN bending states. (C) Real-time blood glucose monitoring with MNs compared with glucose meters and continuous glucose monitoring (CGM) systems. Reproduced with permission from [179]. Copyright 2025, Springer Nature. (D) Schematic of the e-SERS patch configuration. (E) Photograph of the MN patch in practical application. Reproduced with permission from [180]. Copyright 2025, American Chemical Society. (F) Fabrication of FePc–MOF–MX MN sensors. (G) Schematic of in vivo and in vitro detection of L-Cys. Reproduced with permission from [181]. Copyright 2025, Wiley-VCH.

While individual sensors provide specific physiological data, the complex interplay between metabolic and cardiovascular signals necessitates integrated platforms for holistic health management. To achieve this, Chang et al. [179] proposed a biomarkers-linked ultrasound electronic (BLUE), whose structure is shown in Fig. 12A. This system integrates a 3-channel electrochemical MN sensor (detecting glucose, lactate, and ethanol, respectively) with an ultrasonic transducer array, enabling simultaneous monitoring of blood pressure, heart rate, arterial stiffness, and various biochemical indicators. The BLUE system maintained stable performance even after 1,000 repeated tests under wristband bending conditions (Fig. 12B) and showed highly consistent measurement results with commercial devices in real-time glucose monitoring (Fig. 12C). Although the BLUE platform boasts a high degree of functional integration, it still faces challenges such as insufficient MN mechanical durability, high dependence on external circuitry, relatively high power consumption (∼617.92 mW), and a limited variety of detectable biomarkers. Future research needs to further enhance system integration, optimize power management strategies, and promote its validation and application in large-scale clinical environments. Beyond daily health tracking, the rapid pre-hospital diagnosis of acute, life-threatening conditions requires systems capable of capturing both electrophysiological and biochemical signatures simultaneously. For the early diagnosis of acute myocardial infarction (AMI), Yang et al. [180] designed a wearable surface-enhanced Raman scattering (e-SERS) patch, with its structure and application shown in Fig. 12D and E. This patch integrates SERS MNs with flexible electronic components, enabling the simultaneous acquisition of electrocardiogram (ECG) signals and the concentration information of troponin I, myoglobin, and creatine kinase-MB from ISF. The MNs embed DNA “zipper”-type SERS probes, achieving high-sensitivity detection through a competitive adsorption mechanism. The flexible circuit system incorporates a 16-bit analog to digital converter, collecting ECG signals at a sampling frequency of 512 Hz, with an overall power consumption of less than 5 mW. In the AMI rat model, the device detected ECG abnormalities and increased biomarker concentrations within 50 min of ischemia, demonstrating a response capability earlier than traditional serum testing. The patch is waterproof and offers long-term stability, supporting application data visualization, but still has room for development in terms of flexible battery integration and signal analysis algorithm optimization. In parallel with system-level integration, the development of advanced sensing materials is driving the expansion of MN technology toward the detection of specific, oxidation-sensitive biomarkers. Ashraf et al. [181] developed a 3D-printed FePc-MOF-MX MN sensor for the dynamic monitoring of L-cysteine (L-Cys), with its structure and application schematic shown in Fig. 12F. This sensor integrates iron phthalocyanine (FePc), a ZIF-8 type metal–organic framework (MOF), and MXene materials. It exhibits low charge transfer resistance (340 Ω), a wide detection range (0.01 μM to 1 mM), a low detection limit (5 nM), and excellent selectivity, enabling stable operation in serum, urine, and in vivo in mice. Combined with reverse iontophoresis technology, this platform has successfully achieved continuous monitoring of L-Cys in an MI model, demonstrating its application potential in the early diagnosis of cardiovascular and cerebrovascular diseases (Fig. 12G). Nonetheless, anti-fouling capability and sensor stability during long-term use remain limiting factors, which could be optimized in the future through functional coating design or electrochemical regeneration strategies.

##### Microfluidic Analysis

ISF has emerged as a rich reservoir of biomarkers for continuous physiological monitoring; however, relying solely on passive diffusion often limits sampling efficiency and detection sensitivity. To overcome these limitations, recent research has pivoted toward MN-based microfluidic analytical systems, which leverage 3D printing to construct sophisticated fluidic interfaces. By integrating negative pressure mechanisms, vacuum reservoirs, or micro-channels, these systems achieve the active extraction and directed transport of biofluids (ISF or blood), enabling rapid, high-throughput, and multi-modal biochemical analysis (such as colorimetric and immunoassay).

**Fig. 13.**
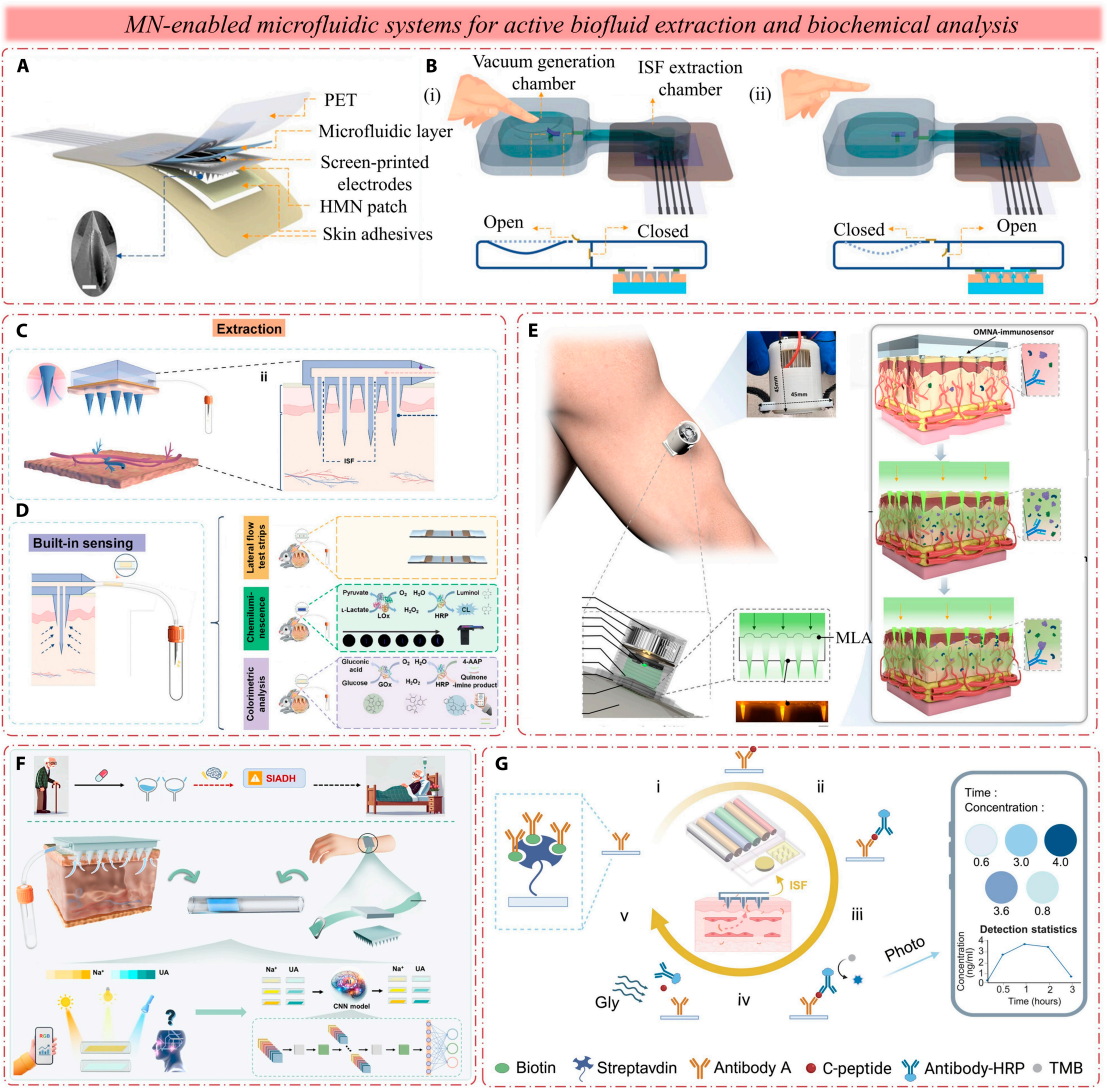
MN-enabled microfluidic systems for active biofluid extraction and biochemical analysis. (A) Schematic of the wearable VIES device based on HMNs. (B) A finger-press negative pressure system leveraging the self-healing properties of PDMS, with dual-valve control enabling continuous ISF extraction via HMNs. Reproduced with permission from [182]. Copyright 2023, Wiley-VCH. (C) Principle diagram of the ISF extraction device. (D) Schematic of the multifunctional sensing platform for rapid ISF analysis. Reprinted from [183] under a CC BY 4.0 license. (E) Photograph of the PIED applied to a human forearm and schematic of its use for blood sampling and multiplexed biomarker detection. Reproduced with permission from [184]. Copyright 2025, Wiley-VCH. (F) Schematic of a CNN-enhanced wearable WMNC for SIADH early warning. Reprinted from [185] under a CC BY 4.0 license. (G) Working mechanism and process of the CIM device. Reproduced with permission from [187]. Copyright 2025, American Association for the Advancement of Science.

To overcome passive diffusion limitations such as low sample volume, integrating active negative pressure mechanisms has become a primary strategy for efficient biofluid acquisition. Building upon this need, Abbasiasl et al. [182] developed a wearable device for vacuum-integrated ISF extraction and sensing (VIES) (Fig. 13A). This system employed a laser-drilled PC HMN array combined with a negative pressure generation unit based on the self-recovering properties of PDMS, enabling ISF extraction via a single press activation without an external pump (Fig. 13B). The MN array could withstand a normal force of 0.4 N/needle and a shear force of 2 N/needle, achieving a penetration depth of approximately 400 μm. The integrated electrochemical sensing module allowed for the simultaneous detection of glucose and pH. This design effectively overcame issues associated with traditional techniques like reverse iontophoresis, such as strong skin irritation, response delay, and sample dilution. While self-powered mechanisms improve portability, maximizing extraction efficiency remains a challenge. To address this, Xie et al. [183] proposed a vacuum tube-integrated 3D-printed HMN array patch (VT-MAP) system, whose extraction principle is illustrated in Fig. 13C. Under a negative pressure of 75 Pa, this device can noninvasively extract 18.42 ± 1.02 μl of ISF from the rabbit ear dermis within 5 min, achieving an extraction efficiency as high as 0.0368 μl/min/needle, which represents the highest reported in vivo extraction efficiency to date. As shown in Fig. 13D, the system integrates colorimetric test strips for the simultaneous analysis of glucose, lactate, and pH, providing a scalable solution for distributed healthcare, although its reusability requires further optimization.

Complementing physical extraction, the integration of optical manipulation and deep learning algorithms has greatly enhanced the sensitivity and accuracy of noninvasive biofluid analysis. Beyond metabolite monitoring, Lin et al. [184] proposed a wearable photothermal-induced extravasation device (PIED) integrating a microlens array (MLA) with optical MNs (OMNA), whose structure and working mechanism are depicted in Fig. 13E. This device employs a low-power light-emitting diode (LED) light source to induce a photothermal effect, expanding local capillaries to promote the extravasation of blood biomarkers into the MN area. Crucially, the MN surface is modified with polyethyleneimine and polyamidoamine, and combined with a biotin-tyramine signal amplification strategy, it achieves a sensitivity for detecting C-reactive protein (CRP) and interleukin-6 (IL-6) that is 6.4 and 7.5 times higher than traditional enzyme-linked immunosorbent assay (ELISA), respectively. The MLA effectively improves the uniformity of light transmission, considerably reducing measurement deviations caused by skin tone differences. Furthermore, the use of medications like desmopressin acetate (DDAVP) for nocturia is associated with risks of nocturnal falls and syndrome of inappropriate antidiuretic hormone secretion (SIADH) [185]. To address these critical safety concerns, Xie et al. [186] proposed a wearable MN colorimetric sensor (WMNC) based on a convolutional neural network (CNN) for the real-time monitoring of Na^+^ and uric acid concentrations. Its structure and early warning mechanism are shown in Fig. 13F. The system utilizes 3D printing to fabricate a dual-channel HMN for ISF sampling combined with vacuum negative pressure, and detects the targets via enzyme-catalyzed colorimetric reactions. To ensure reliable readouts in complex lighting environments, the study introduced a CNN model for image recognition, eliminating the reliance on specialized detection light sources. Animal experiment results demonstrated that the device could accurately track the concentration changes of Na^+^ and uric acid after DDAVP administration, showing high consistency with blood test results. Besides, continuous C-peptide monitoring is pivotal for precise diabetes classification and dynamic management. Addressing this, Chen et al. [187] developed a wearable device based on continuous immunoassay monitoring (CIM) for the noninvasive continuous monitoring of C-peptide (Fig. 13G). This system extracts ISF using HMNs and negative pressure suction, and employs a double-antibody sandwich method for C-peptide detection. The device integrates a microfluidic reaction chamber and a smartphone platform, completing the detection process within 15 min, and revealed species-specific differences in C-peptide concentrations between blood and ISF. Future research needs to further improve the integration of wireless modules and delve deeper into the transport mechanisms of biomarkers between ISF and blood.

#### MN-enabled field-assisted and feedback interventions

##### Field-assisted Actuation

While the diagnostic platforms described in the preceding subsection excel at high-fidelity information acquisition, comprehensive clinical management necessitates a transition from passive observation to active tissue modulation. Consequently, this section highlights the evolution of MN systems from passive delivery interfaces to active therapeutic platforms enabled by field-assisted actuation and feedback control mechanisms. By harnessing external physical fields, such as acoustic, electrical, or mechanical energy, or integrating intelligent closed-loop algorithms, these systems achieve precise, on-demand, and personalized interventions. This shift empowers MNs to overcome biological barriers and regulate physiological states dynamically, representing a clear trend toward portable, intelligent, and functionally unified minimally invasive devices in clinical applications.

**Fig. 14.**
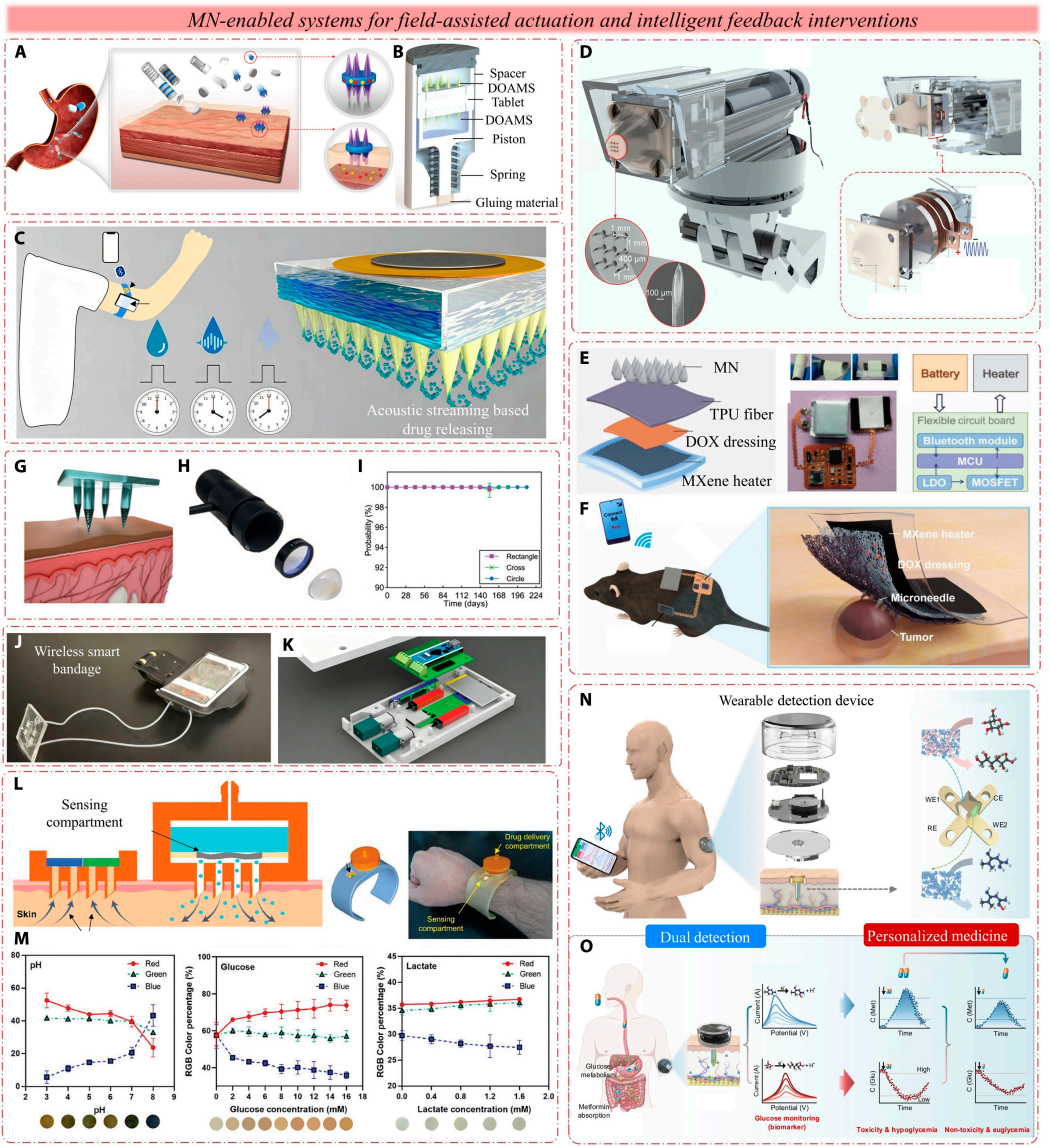
MN-enabled systems for field-assisted actuation and intelligent feedback interventions. (A) Schematic of the DOAMS autonomously responding to low pH in the stomach and deploying engineered tablets into tissue. (B) Schematic of the “jack-in-the-box” capsule. Reprinted from [188] under a CC BY 4.0 license. (C) Schematic of an acoustically actuated, programmable, wearable patch based on MN arrays. Reproduced with permission from [189]. Copyright 2023, Elsevier. (D) Schematic of a PDMN platform. Reproduced with permission from [113]. Copyright 2024, Cell Press. (E) Schematic of a stretchable electronic patch and its integration with a circuit system. (F) Schematic of a stretchable MN-integrated electronic patch for wearable cancer therapy. Reproduced with permission from [190]. Copyright 2025, Wiley-VCH. (G) MN arrays deliver fluorescent microparticles into the skin to create spatially encoded patterns enabling noninvasive imaging readout of vaccination status. (H) Schematic of a smartphone with the internal short-pass filter removed and an 850-nm long-pass filter installed. (I) Illustration of average probability output from a machine learning algorithm. Reproduced with permission from [191]. Copyright 2019, American Association for the Advancement of Science. (J) Schematic of a wireless smart bandage based on 3D-printed MN arrays. (K) Schematic of the microcontroller comprising a drug reservoir, micropump, power supply, and electronic circuitry. Reproduced with permission from [192]. Copyright 2020, Wiley-VCH. (L) Diagram of the MNA system. (M) Schematic of quantitative detection of pH, glucose, and lactate. Reproduced with permission from [111]. Copyright 2024, Wiley-VCH. (N) Schematic of the MCBM system. (O) Regulation of blood glucose and drug concentration simultaneously within normal range via the MCBM platform. Reproduced with permission from [193]. Copyright 2025, Springer Nature.

While most MN systems focus on transdermal pathways, 3D printing technology is also revolutionizing oral biologics delivery by enabling sophisticated mechanical interlocking structures to overcome mucosal barriers. To address the challenges of intestinal enzymatic degradation and low absorption rates, Chen et al. [188], inspired by the intestinal attachment mechanism of thorny-headed intestinal worm, designed a dynamic omnidirectional adhesive MN system (DOAMS) (Fig. 14A). This system employs a Carbopol hydrogel shell combined with a PCL core structure, offering both strong adhesion and mechanical stability. In a porcine stomach model, the DOAMS demonstrated excellent tissue adhesion and deformation recovery capabilities. Combined with a self-triggering capsule for delivering semaglutide and the absorption enhancer sodium N-[8-(2-hydroxybenzoyl) amino] caprylate (SNAC), this system markedly improved the oral bioavailability of the drug (Fig. 14B). However, its long-term tissue compatibility, reusability stability, and material degradation behavior still require systematic investigation. Although mechanical structures solve retention issues, precise dosage regulation in transdermal systems requires active driving mechanisms to surpass the limitations of passive diffusion. Addressing this, Wu et al. [189] proposed an acoustically actuated programmable MN patch (Fig. 14C). This system integrates an MN array with a piezoelectric ceramic transducer (PZT), a microfluidic drug reservoir, and control circuitry. It utilizes 34-kHz acoustic waves to induce vortex effects for active drug pumping, achieving a peak flow rate of 40 μl/min with precisely controllable release profiles. The system demonstrates good biocompatibility and promotes skin repair; however, its portability, level of circuit integration, and adaptability for delivering various drugs still require improvement. Beyond fluid pumping, physical fields can also be harnessed to deepen tissue penetration for enhanced cancer therapy. In photodynamic therapy (PDT), the limited penetration depth of traditional photosensitizers restricts the generation of sufficient ROS in deep lesions, severely constraining therapeutic efficacy. Chen et al. [113] developed a piezoelectric-driven MN (PDMN)-based PDT platform (Fig. 14D). The system employed 3D printing to fabricate multi-channel MNs and combined them with ultrasonic cavitation effects to achieve high-efficiency delivery of the photosensitizer to the dermis (up to 1.5 mm deep) and enhanced ROS generation. This substantially improved the clinical efficacy of PDT in diseases such as viral warts, mastopathy, and skin cancer. Complementing acoustic strategies, electrothermal actuation offers a versatile modality for triggering on-demand drug release in wearable oncology. Yang et al. [190] proposed a stretchable electronic patch integrated with PMNs for chemo-thermal combination therapy. Its structure and application are shown in Fig. 14E and F, respectively. This MN patch integrates phase-change microcarriers loaded with DOX and an MXene-based heater, maintaining electrothermal stability under mechanical strain and supporting remote, personalized treatment control. Animal experimental results demonstrated that this system greatly inhibited melanoma growth, exhibiting excellent synergistic therapeutic effects and promising potential for clinical translation.

##### Feedback Control

In the field of vaccine delivery and information recording, McHugh et al. [191] developed a DMN patch capable of simultaneously delivering a vaccine and NIR quantum dots (QDs), creating an invisible vaccination record system (Fig. 14G). This system can penetrate to a depth of 750 μm in porcine skin, and the implanted QD pattern can be recognized with high accuracy using a modified smartphone combined with a machine learning algorithm, with the signal remaining stable for over 9 months (Fig. 14H and I). This platform offers advantages such as low cost and independence from cold chain storage, making it suitable for widespread vaccination needs in resource-limited settings. However, the stability of the QDs coexisting with the vaccine and their biocompatibility still require in-depth investigation. Beyond static information storage, the integration of active control systems enables the programmable management of complex, dynamic conditions like chronic wounds. Derakhshandeh et al. [192] developed a wireless smart bandage system integrating HMNs and a micropump for the programmable treatment of chronic wounds (Fig. 14J). This system enables the deep delivery of multiple drugs through HMNs, effectively overcoming the issue of reduced drug efficacy associated with traditional dressings caused by necrotic tissue barriers and exudate washout. As shown in Fig. 14K, the platform features a modular design, integrating a micropump, wireless control, and a flexible substrate, allowing physicians to remotely regulate the dosage and timing of drug release via a smartphone to implement personalized treatment strategies. Similarly, the integration of sensing and delivery modules facilitates telemedicine applications, allowing remote therapeutic management. Razzaghi et al. [111] fabricated an HMN array based on DLP 3D printing technology, constructing a remotely operated integrated platform (Fig. 14L). The design utilized a 45° bevel angle to optimize penetration capability and integrated a paper-based colorimetric sensor to achieve real-time quantitative detection of pH, glucose, and lactate in the ISF (Fig. 14M). The drug delivery component, driven by ultrasonic atomization, demonstrated higher efficiency than classical transdermal techniques. This system supports smartphone control for preliminary closed-loop therapeutic management, but further reinforcement is needed in expanding the range of detectable biomarkers and validating long-term safety. Furthermore, to achieve real-time assessment of drug efficacy and closed-loop feedback control in diabetes treatment, Yang et al. [193] developed an MN-based continuous biomarker/drug monitoring (MCBM) system, whose structure and working principle are illustrated in Fig. 14N. This system integrates a dual-functional electrochemical sensor that utilizes differential pulse voltammetry for the highly sensitive detection of both glucose (0 to 28 mM) and metformin (0 to 140 μM) in the skin ISF. The MN features a 4-channel electrode configuration and is combined with a wireless circuit and a smartphone application, enabling real-time data processing and adaptive drug dosage adjustment. Furthermore, the system can simultaneously monitor the dynamics of blood glucose and drug concentrations, constructing individualized pharmacokinetic models to maintain both parameters stably within the therapeutic window (Fig. 14O). Although the system demonstrates good biocompatibility and mechanical properties, its long-term stability, cost control, and broader application in chronic disease management still require further investigation.

This section provides a systematic review of recent advancements in integrated MN systems, encompassing 2 primary domains: MN-enabled bioelectronic and microfluidic diagnostics, and field-assisted and feedback interventions. By integrating MNs with bioelectronic and microfluidic interfaces, as well as physical field actuators and feedback circuits, these systems enable the high-fidelity digitization of physiological signals and the execution of precise active interventions. To comprehensively evaluate the design principles and technical features of these integrated systems, their key attributes are detailed systematically in Table 5. This comparison clearly illustrates that integrated MN systems are accelerating the evolution of minimally invasive biomedical devices toward greater portability, intelligence, and functional unification.

**Table 5.** A comparative analysis of 3D-printed integrated MN systems for biomedical applications

Types	3D printing method	MN type	MN structure	MN material	Therapeutic agents	Integration	Advantages	Application	Ref.
MN-enabled bioelectronic and microfluidic diagnosis (bioelectronic sensing)	DLP	SMNs	H 0.5-1.5 mm, D_b 0.1-0.4 mm	Clear/biocompatible resin	/	PB-Au WE, Ag/AgCl RE	Painless, continuous	ISF glucose monitoring	[174]
	TPP	SMNs	H 600 μm, D_b 400 μm	OrmoStamp + Au coating	/	Au, PL	Precise, stable, cost-efficient	Biosensing	[175]
	DLP	HMNs	Pyramid, H 1,000 μm, D_b 1,000 μm	Acrylic resin	/	PEDOT: PSS filled	High-res temp, robust	Intradermal temp monitoring	[176]
	DLP	SMNs	Pyramid	PAMS hydrogel	/	With PCB, knee guard	Sensitive, antimicrobial	Knee microstrain monitoring	[178]
	SLA	SMNs	H > 800 μm, Tip D 5–10 μm	SU-8 photoresist + Cr/Pt coating	/	US and electrochem sensors	Multimodal, minimal crosstalk	Diabetes/Cardio monitoring	[179]
	PμSL	HFMNs	Conical array, H 600 μm, D_b 300 μm	HAMA/PEGDA hydrogel	/	Flexible electronics	Ultra-sensitive, real-time	AMI diagnosis	[180]
	LCD MSLA	SMNs	Conical, H: 1,000 μm, D_b 250 μm	Biocompatible resin (surgical guide)	/	Three-electrode system	Micro-invasive, sensitive	L-Cys monitoring (MI)	[181]
MN-enabled bioelectronic and microfluidic diagnosis (microfluidic analysis)	PμSL	HMNs	Pyramid, H 1.5 mm, W 0.6 mm	PC	/	Vacuum, microfluidics, BT	Single-touch vacuum, anti-clog	ISF glucose/pH sampling	[182]
	SLA	Dual-channel HMNs	Conical, H 1,000 μm, Channel D 250 μm	PMA resin	/	On-board analysis	Efficient ISF sampling	Home health monitoring	[183]
	3D-printed mold	Optical SMNs	Conical, H≈600 μm, D≈100 μm	PMMA	/	LED, MLA, temp sensor	Noninvasive, universal	CRP/IL-6 detection	[184]
	SLA	Dual-channel HMNs	Conical tip, H 1,000 μm, 10x10 array	PMA resin	/	Vacuum, paper sensor	Wearable, efficient	Na^+^/uric acid monitoring	[186]
	SLA	Dual-channel HMNs	6x6 array, H 650 μm, Dual channels (D 150 μm)	PMA resin	/	PDMS chip, vacuum	Strong, renewable	C-peptide diagnosis	[187]
MN-enabled field-assisted and feedback interventions	TPP	DMNs	Cylinder-Cone, Total H 1,500 μm, D_b 300 μm	PVA/sucrose + PMMA (for QDs)	IPV2	NIR QDs, smartphone	Cold-chain free, stable	Vaccination record	[191]
	FDM	HMNs	H 2 mm, Spacing 1.5–3 mm, Orifice D 0.2–0.5 mm	VeroClear, TangoBlack	VEGF, antibiotics (e.g., cefazolin)	PDMS, micropumps, wireless	Targeted, programmable	Chronic wound therapy	[192]
	SLA	CSMNs	Conical, H 1.3 mm	Shell: Carbopol 971P NF, core: PCL	Semaglutide, SNAC	Spring-piston, trigger	Adhesive, triggered release	Oral GLP-1 delivery	[188]
	PμSL	HMNs	10x10 array, H 1,000 μm, Inner D 200 μm	BIO resin	Liquid agents (e.g., sodium fluorescein)	PZT, BT	Programmable, acoustic	Transdermal delivery	[189]
	DLP	HMNs	45° needle angle, Sensing port D 400 μm, Delivery port D 200–500 μm	PEGDA 250, Irgacure 819, Sudan I	Small molecules, biomolecules (e.g., B27)	Colorimetric paper, nebulizer	Remote, pump-free	On-demand delivery	[111]
	PμSL	Multi-channel HMNs	Outer D 185 μm, Inner D 120 μm, H 1 mm, 3x3 array	/	MB, 5-ALA	PZT, electrode	Enhanced drug permeation	Skin PDT	[113]
	DIW	PMNs	H 600 μm, D_b 400 μm, tip < 8 μm, porosity 42.9%	Cellulose acetate/SiO_2_ NPs	DOX	MXene, wireless circuit	Stretch-insensitive, wireless	Tumor therapy	[190]
	PμSL	SMNs	H 2 mm, W 900 μm, 4 microchannels	BIO resin + Au/Pt film	/	PCB, BT, App	Dual-analyte, real-time	Glucose/metformin monitoring	[193]

D_b, base diameter/base width; D, diameter; W, width; 5-ALA, 5-aminolevulinic acid; BIO resin, a biocompatible photopolymer resin; Carbopol 971P NF, a cross-linked poly(acrylic acid) polymer; Cr, chromium; DIW, direct ink writing; IPV2, inactivated poliovirus vaccine type 2; MB, methylene blue; PMA, poly(methacrylate); Pt, platinum; SiO_2_ NPs, silicon dioxide nanoparticles; BT, Bluetooth; Cardio, cardiovascular; GLP-1, glucagon-like peptide-1; PCB, printed circuit board; US, ultrasound; WE, working electrode; RE, reference electrode

## Conclusion and Outlook

**Fig. 15.**
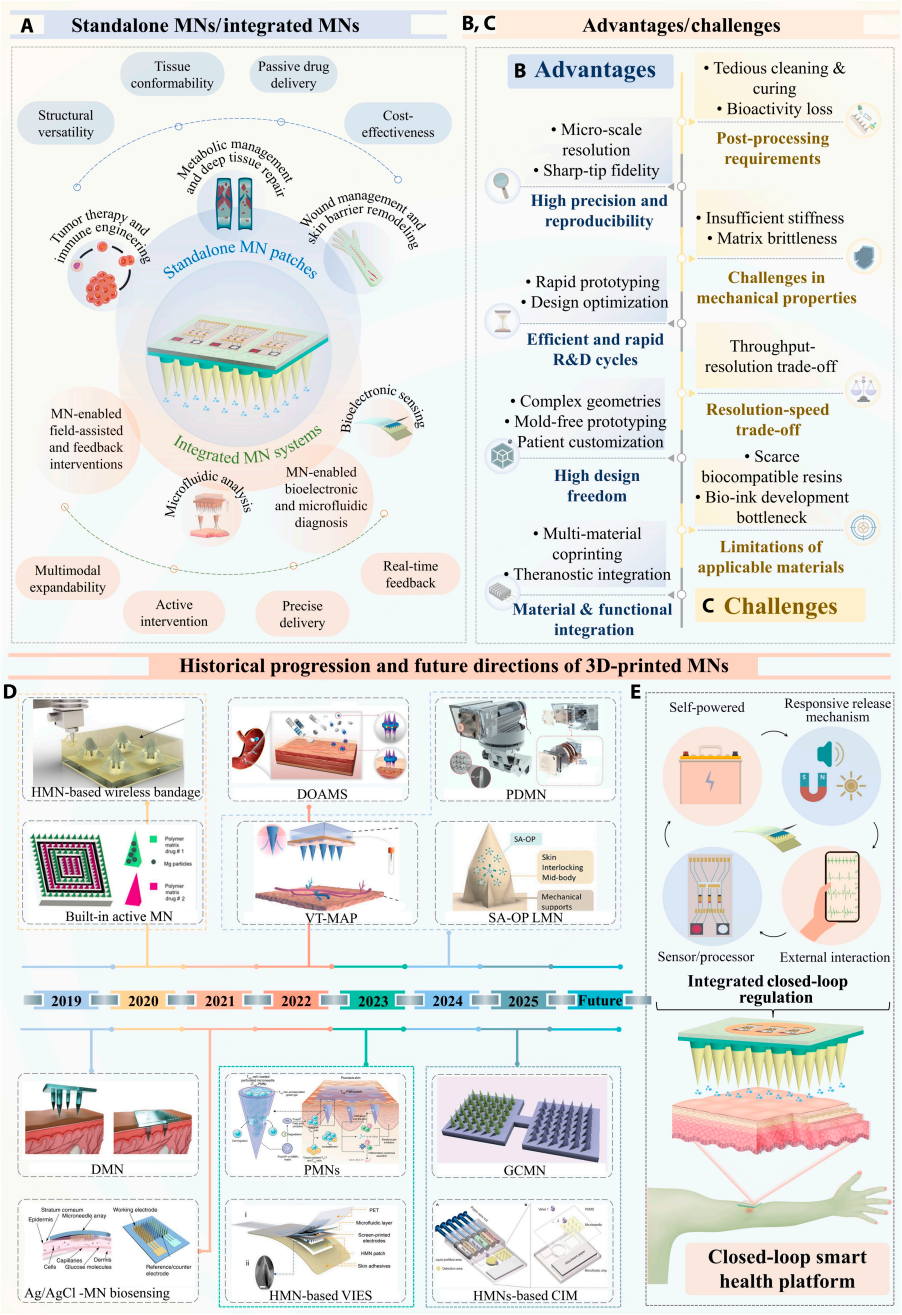
Technology landscape and future outlook of 3D-printed MNs. (A) Application and scenarios for standalone and integrated MN systems. (B) Advantages of 3D-printed MNs. (C) Key challenges and strategies for clinical translation. (D and E) Developmental trajectory and future directions of 3D-printed MNs. (D) From microstructural fabrication to intelligent integration. Reproduced with permission from [106,113,158,163,167,174,182,183,186,188,191,192]. Copyright 2019, American Association for the Advancement of Science; 2020, Wiley-VCH; 2021, Springer Nature; 2022, American Association for the Advancement of Science; 2022, American Chemical Society; 2023, Wiley-VCH; 2023, American Association for the Advancement of Science; 2024, Cell Press; 2024, Wiley-VCH; 2025, American Association for the Advancement of Science; 2025, Wiley-VCH. (E) Toward a closed-loop smart health platform.

Driven by clinical needs, 3D-printed MNs are rapidly diversifying in biomedicine, evolving into 2 distinct but complementary functional domains: standalone MN patches and integrated intelligent systems (Fig. 15A). Standalone patches focus on multi-dimensional therapy, including superficial skin repair, systemic metabolic regulation, and targeted tumor treatment, enabled by optimized geometric designs and material strategies. Their advantages are simplicity, cost-effectiveness, and ease of deployment. Conversely, integrated MN systems represent a higher stage of evolution, categorized into bioelectronic and microfluidic diagnostics for high-fidelity information acquisition, and field-assisted and feedback interventions for active therapy. These systems co-design sensors, microfluidics, and actuation modules to establish closed-loop platforms. Accordingly, the 2 approaches are mutually reinforcing, collectively expanding MNs in personalized medicine. The foundational driver of this diverse landscape is the unparalleled freedom 3D printing offers in structural design, material integration, and functional realization (Fig. 15B). It overcomes the limitations of conventional techniques in constructing complex geometries and enables patient-specific customization via digital model-based fabrication. Furthermore, by enabling “one-step molding”, it achieves the high-level integration of diverse functional materials, including conductive polymers, stimuli-responsive hydrogels, and drug-loaded polymers, simplifying fabrication and accelerating the development of smart MN patches. Notably, advanced MN systems developed via conventional approaches, such as magnetic MN array robots and MXene-integrated hydrogel MNs [194,195], could also be enhanced by 3D printing. Patient-specific gradient structures and magnetic responsiveness can be fabricated more directly, while multi-material encapsulation and thermoresponsive release may be streamlined through monolithic integration of drug reservoirs and photothermal layers in a single step, thereby improving reproducibility and scalability for complex, multi-component biomedical platforms.

However, despite these technological strides, the transition of 3D-printed MNs from laboratory research to large-scale clinical translation faces a series of formidable challenges (Fig. 15C). To address these issues and guide future developments, the following core research directions and challenges should be prioritized:

Advancing manufacturing resolution and structural fidelity: A fundamental challenge lies in the trade-off between printing resolution and build speed: high-precision techniques are often expensive and time-consuming, whereas faster methods still struggle to achieve fine microstructures. Furthermore, the high-fidelity reconstruction of complex architectures with extreme overhangs remains a major bottleneck for many 3D printing techniques, as these features often require auxiliary support structures that are difficult to remove at the micro-scale without compromising needle integrity. Future research should focus on developing hierarchical printing strategies, volumetric 3D printing strategies, multi-scale hybrid fabrication approaches, and optimized adaptive slicing and path-planning algorithms to enhance the local resolution of critical features.

Developing biofunctional and mechanically robust material systems: Current photopolymerization-based processes rely on photosensitive resins that require rigorous long-term evaluation for biocompatibility, biodegradability, and photoinitiator toxicity. Moreover, since many ideal pharmaceutical polymers lack intrinsic printability, the development of novel bioinks combining excellent print fidelity with superior biological performance is urgently needed. Mechanically, most printable polymers and high-water-content hydrogels suffer from low mechanical strength, insufficient skin penetration capability, or brittleness, which hampers their ability to effectively breach the stratum corneum. Solutions involve developing high strength printing materials, enhancing stability through nanoparticle reinforcement, structural optimization, and the application of lubricating coatings.

Standardization and regulatory frameworks for clinical translation: Efficient post-processing remains a bottleneck; achieving controlled cleaning, curing, surface modification, and drug coating while preserving the activity of functional molecules will be a central focus of future engineering. Crucially, for successful clinical translation, it is imperative to establish standardized AM protocols, implement robust safety assessment frameworks, and adapt regulatory systems to accommodate emerging biomaterial technologies. Functionally, the use of multi-material coprinting and optimized interface engineering is expected to facilitate the unification of diagnostic and therapeutic functions within a single monolithic device.

Evolution toward integrated and closed-loop smart platforms: Aligned with the visionary trend of MNs at the forefront of theranostics [196], 3D-printed MNs are spearheading a paradigm shift from the traditional “penetration–delivery” model to an integrated “monitoring–analysis–feedback–intervention” closed-loop regulation (Fig. 15D and E). This evolution represents a fundamental transition from static microstructural construction to mechanism-oriented device orchestration, bridging the gap between descriptive morphology and functional logic. Within this context, the field is transcending traditional 3D architectures toward 4D printing, enabling MNs to dynamically evolve their morphology or function in response to the physiological microenvironment, thereby granting them the “active” responsive capabilities required for autonomous intervention. Conceptually, the future lies in bioelectronic therapeutic platforms that seamlessly integrate sensing elements, processing units, and wireless modules. To achieve true precision in these complex systems, the integration of Digital Twin-assisted design will be essential, allowing for the virtual simulation and predictive optimization of patient-specific therapy before and during administration.

Although challenges such as biocompatibility verification and long-term stability remain, the trajectory suggests that 3D-printed MNs will become a key technological enabler in advancing medical devices toward greater intelligence, multifunctionality, and personalization, ultimately redefining the future of precision healthcare.

## Data Availability

The data that support the findings of this study are available from the corresponding authors upon reasonable request.
